# Advancements in Plant-Based Therapeutics for Hepatic Fibrosis: Molecular Mechanisms and Nanoparticulate Drug Delivery Systems

**DOI:** 10.3390/ijms25179346

**Published:** 2024-08-28

**Authors:** Alina Ciceu, Ferenc Fenyvesi, Anca Hermenean, Simona Ardelean, Simona Dumitra, Monica Puticiu

**Affiliations:** 1 “Aurel Ardelean” Institute of Life Sciences, Vasile Goldis Western University of Arad, 86 Rebreanu, 310414 Arad, Romania; ciceu.alina@uvvg.ro; 2 Department of Molecular and Nanopharmaceutics, Faculty of Pharmacy, University of Debrecen, 4032 Debrecen, Hungary; fenyvesi.ferenc@pharm.unideb.hu; 3 Faculty of Pharmacy, Vasile Goldis Western University of Arad, 86 Rebreanu, 310414 Arad, Romania; 4 Faculty of Medicine, Vasile Goldis Western University of Arad, 86 Rebreanu, 310414 Arad, Romania; dumitra.simona@uvvg.ro (S.D.); puticiu.monica@uvvg.ro (M.P.)

**Keywords:** phenolic acids, flavonoids, hepatic fibrosis

## Abstract

Chronic liver injuries often lead to hepatic fibrosis, a condition characterized by excessive extracellular matrix accumulation and abnormal connective tissue hyperplasia. Without effective treatment, hepatic fibrosis can progress to cirrhosis or hepatocellular carcinoma. Current treatments, including liver transplantation, are limited by donor shortages and high costs. As such, there is an urgent need for effective therapeutic strategies. This review focuses on the potential of plant-based therapeutics, particularly polyphenols, phenolic acids, and flavonoids, in treating hepatic fibrosis. These compounds have demonstrated anti-fibrotic activities through various signaling pathways, including TGF-β/Smad, AMPK/mTOR, Wnt/β-catenin, NF-κB, PI3K/AKT/mTOR, and hedgehog pathways. Additionally, this review highlights the advancements in nanoparticulate drug delivery systems that enhance the pharmacokinetics, bioavailability, and therapeutic efficacy of these bioactive compounds. Methodologically, this review synthesizes findings from recent studies, providing a comprehensive analysis of the mechanisms and benefits of these plant-based treatments. The integration of novel drug delivery systems with plant-based therapeutics holds significant promise for developing effective treatments for hepatic fibrosis.

## 1. Introduction

Chronic liver injuries are a primary manifestation of hepatic fibrosis [1], which represents an abnormal wound healing response characterized by excessive extracellular matrix (ECM) accumulation and abnormal connective tissue hyperplasia [2]. Without effective treatment, hepatic fibrosis can advance to cirrhosis or hepatocellular carcinoma [3]. Currently, liver transplantation is the most effective treatment for cirrhosis, but its clinical application is limited due to the shortage of donors and high costs [4]. There is no specific medication for treating hepatic fibrosis, and many hepatic anti-fibrotic drugs are still in the research and development phase [1]. Considering the severe consequences of hepatic fibrosis, understanding the underlying mechanisms leading to its development and progression is crucial. This understanding is essential for developing effective therapeutic strategies [2].

Polyphenols are increasingly gaining attention for the development of potential drugs for liver disease treatment. Numerous polyphenols have demonstrated hepatic anti-fibrotic activity by inhibiting the activity of hepatic stellate cells (HSCs) [2]. These bioactive compounds operate through various pathways, including the TGF-β/Smad signaling pathways, AMPK/mTOR, Wnt/β-catenin, NF-κB, PI3K/AKT/mTOR, hedgehog pathways, and other factors associated with hepatic fibrosis.

Hepatic fibrosis can be mitigated using medicinal plants, plant extracts, and bioactive compounds derived from plants that inhibit the activation of hepatic stellate cells and reduce ECM deposition [5,6]. Plant extracts are a mixture of bioactive compounds and pharmacokinetic synergists [7]. These biologically active compounds can work synergistically to enhance the therapeutic efficacy of plant-based medicines [8]. Medicinal plants and their phytocompounds can protect the liver through various mechanisms, including the inhibition of fibrogenesis, oxidative stress, and tumor growth [9].

As most liver injuries are chronic conditions that require long-term treatment, it is important to minimize the side effects of hepatoprotective drugs. All bioactive compounds, including plant-based drugs, can have adverse effects. Therefore, further research on plant-based drugs with hepatic anti-fibrotic effects is necessary [10]. Despite significant progress in understanding the pathogenesis of hepatic fibrosis, no effective agent has been developed yet to prevent or directly reverse the fibrotic process [11]. The administered dose of biologically active compounds significantly influences the clinical response. Higher doses of such compounds have shown superior clinical efficacy but are associated with increased toxicity in various organs [12]. Many plant-based drugs and plant extracts have poor absorption and low bioavailability due to their poor lipid solubility or improper molecular sizes [13].

The aim of this review is to comprehensively analyze and synthesize current research on the anti-fibrotic effects of polyphenols, specifically phenolic acids and flavonoids, and to evaluate the advancements in nanoparticulate drug delivery systems that enhance the pharmacokinetics and therapeutic efficacy of these bioactive compounds. By exploring the molecular mechanisms through which polyphenols modulate key signaling pathways implicated in hepatic fibrosis, and by assessing the potential of novel delivery systems to improve their bioavailability and reduce side effects, this review seeks to provide a detailed understanding of the potential therapeutic applications in the treatment of hepatic fibrosis and to identify future research directions in this field.

## 2. Polyphenols and Hepatic Fibrosis

The intricate mechanisms driving hepatic fibrosis highlight the need for combined therapeutic approaches that target multiple signaling pathways. In addition to chemical compounds, various natural products have shown effectiveness in treating hepatic fibrosis [14]. Polyphenols, which are secondary metabolites naturally found in many plant-derived foods and beverages commonly consumed in the human diet, are particularly noteworthy. Based on their chemical structure, polyphenols are classified into several categories: phenolic acids (including hydroxycinnamic and hydroxybenzoic acids), flavonoids, stilbenes, tannins, and lignans [15,16,17,18,19].

### 2.1. Phenolic Acids

Phenolic acids are the simplest phenolic compounds, characterized by a single phenolic ring with multiple hydroxyl or methoxyl groups attached [20]. They are divided into two main categories: derivatives of hydroxycinnamic acid and derivatives of hydroxybenzoic acid [21].

Hydroxycinnamic acids are aromatic carboxylic acids with an unsaturated side chain [22]. In these acids, the carboxylic acid functional group is separated from the phenol ring by a double bond (C=C) [21]. Cinnamic acids function as phytohormones and are precursors to chalcones, flavonoids, anthocyanins, and stilbenes [22]. Hydroxycinnamic acids associated with hepatic fibrosis include chlorogenic acid, ferulic acid, isochlorogenic acid, p-coumaric acid, rosmarinic acid, salvianolic acids A and B, and sinapic acid (Table 1).

Hydroxybenzoic acids are phenols substituted with a carboxylic acid functional group directly bonded to the phenol ring [21]. These acids are less abundant in plants and are components of complex structures such as tannins and lignins [23]. This category includes p-hydroxybenzoic, protocatechuic, vanillic, syringic, and gallic acids [24]. Hydroxybenzoic acids with hepatic anti-fibrotic activity include gallic acid, protocatechuic acid, and vanillic acid (Table 1).

**Table 1:** Pharmacological effects of phenolic acids in liver fibrosis.

Class of Phenolic Acids	Bioactive Compounds	Cell Lines/ Animal Model	Pharmacological Effects	Reference
Hydroxycinnamic acids	Chlorogenic acid	LX-2 cells Sprague-Dawley rats	- inhibited the mRNA expression of miR-21, CTGF, α-SMA, TIMP-1, and TGF-β1 and the protein expression of p-Smad2, p-Smad3, p-Smad2/3, CTGF, α-SMA, TIMP-1, and TGF-β1 both in vitro and in vivo - ↑ the mRNA and protein expression of Smad7 and MMP-9 - ↓ the degree of liver fibrosis - ↓ α-SMA and Col-1 expression in liver tissue - ↓ TGF-β1 in serum	[25]
		Sprague-Dawley rats	- attenuated CCl_4_-induced liver damage - ↓ ALT, AST - ↑ ALB - alleviated the degree of liver fibrogenesis and formation of pseudo-lobulus - ↓ α-SMA and Col-1 - ↓ the expression levels of TLR4, MyD88, iNOS, and COX-2 - ↑ BAMBI - suppressed CCl_4_-induced NF-κB activation - ↓ hepatic mRNA expression and serum levels of TNF-α, IL-6, and IL-1β	[26]
	Ferulic acid	MPHs, RAW 264.7 cells, and LX-2 cells C57BL/6J mice	- ameliorated CCl_4_-induced inflammation and fibrotic liver damage in mice - ↓ ALT, AST - ↓ MDA - ↑ SOD - ↓ collagen deposition - ↓ fibronectin, Col-1, TGF-β, Acta2 - ↑ the phosphorylation of AMPK and ERK1/2 - inhibited hepatic oxidative stress, macrophage activation, and HSC activation via AMPK phosphorylation in different liver cells - ↓ MDA, NOX2 - ↑ SOD - ↓ ROS production in MPHs - ↓ IL-1β, F4/80, and Cd11b - ↓ pro-inflammatory gene transcript levels of Ccl2 and TNF-α, IL-6, and iNOS - promoted the translocation of NF-κB P50 and P65 from nucleus to cytoplasm - inhibited the activity of PTP1B	[27]
	Isochlorogenic acid B	C57BL/6 mice	- improved the pathological lesions of liver fibrosis - ↓ serum ALT, AST, HYP, cholesterol, triglycerides - inhibited HSC activation - ↓ the expressions of hepatic genes involved in liver fibrosis: LOX, TGF-β1, MCP-1, Col1α1, TIMP-1 - attenuated liver oxidative stress through Nrf2 signaling pathway	[28]
	p-Coumaric acid	LX-2 cells C57BL/6 mice	- improved systemic insulin sensitivity without altering adiposity - ↓ ALT, AST - attenuated hepatic signaling pathways associated with NLRP3 inflammasome activation: TLR4/NF-κB, and endoplasmic reticulum/oxidative stress - ↓ circulating IL-1β levels - ameliorated hepatic fibrosis - ↓ the excessive deposition of collagen fibers - ↓ α-SMA - normalized the expression of TGF-β, Col1α2, Col3α1, Col4α1, and TIMP-1 - ↓ NLRP3 activation and caspase-1 cleavage	[29]
	Rosmarinic acid	Sprague-Dawley rats HSC-T6	- inhibited HSC proliferation - inhibited TGF-β1, CTGF, and α-SMA expression in cultured HSCs - ↓ collagen deposition - ameliorated hepatocyte degeneration, necrosis, and infiltration of inflammatory cells - ↓ serum levels of HA, LN, and PCIII - ↑ ALB/GLB - ↓ ALT, AST, HYP - inhibited TGF-β1, CTGF expression in vivo	[30]
	Salvianolic acid A	Sprague-Dawley rats	- ↓ liver fibrosis by inhibiting liver function, liver fibrosis index, and collagen deposition in vivo - ↓ ALT, AST, HA, CIV, LN, PIIIP - ↓ HYP - ↓ α-SMA, TGF-β1, PDGF-βR, CTGF, desmin, and vimentin - inhibited the PI3K/AKT/mTOR signaling cascade - ↓ p-AKT, p-mTOR, p-p70S6K1 - prevented the stimulation of hepatic stellate cells and the synthesis of ECM - ↓ the hepatocyte apoptosis - ↑ Bcl-2 - ↓ Bax - ↓ caspase-3 and cleaved caspase-3	[31]
	Salvianolic acid B	C57BL/6 mice LO2 cells	- attenuated liver fibrosis in CCl_4_-induced mice - ↓ ALT, AST - ↓ the hepatic inflammatory cell infiltration - ↓ collagen levels - alleviated liver fibrosis in mice by targeting up-regulation of Ecm1 and inhibiting hepatocyte ferroptosis	[32]
		C57BL/6 mice LX2 and WRL68 cells	- alleviated hepatic fibrogenesis by inhibiting the activation of HSCs and collagen deposition - ↓ ALT, AST - displayed anti-inflammatory effects in CCl_4_-induced liver fibrosis - ↓ IL-1β, IL-6 - ↓ the infiltration of CD68 and CD11b cells in liver - ↓ γH2AX	[33]
		HSC-LX-2 cells BALB/c mice	- had a good binding ability to PDGFRβ - inhibited the activation of HSCs in vitro - ↓ the mRNA expression levels of α-SMA and Col-1 - attenuated HSC activation by targeting PDGFRβ pathways - ↓ p-AKT/AKT, p-ERK/ERK, and p-p38/p38 signaling pathways - inhibited the migration and proliferation and promoted apoptosis of HSCs - suppressed PDGF-BB-induced HSC activation and the PDGFBB/PDGFRβ pathway in vitro - ↓ α-SMA and Col-1 - ↓ the expression of p-PDGFRβ/PDGFRβ, p-AKT/AKT, p-ERK/ERK, and p-p38/p38 proteins - inhibited PDGFRβ signaling pathway, HSC activation improved CCl_4_-induced liver fibrosis and inflammation in vivo - ↓ ALT, AST - ↓ α-SMA and Col-1 - ↓ the expression of p-AKT, p-ERK, and p-p38 proteins - ↓ the mRNA expression levels of inflammatory factors IL-1β, IL-6, TNF-α, TGF-β, and COX-2	[34]
		JS1 and LX2 cells	- inhibited autophagy of HSCs induced by TGF-β1 - inhibited the protein expression of LC3B II - induced the expression of C-Caspase 3 - inhibited activation of JS1 through repressing autophagy of JS1 induced by TGF-β1 - ↓ LC3B II, Atg5, α-SMA, and Col-I protein expressions - inhibited activation and autophagy of HSCs by down-regulating the ERK, p38, and JNK pathways - ↓ p-ERK, p-JNK, and p-p38 MAPK protein expressions	[35]
		Sprague-Dawley rats	- inhibited the CCl_4_-induced histopathological deterioration of the liver - ↓ α-SMA - ↓ liver damage caused by CCl_4_ - ↓ serum ALT, AST, TBIL - ↑ ALB - ↓ TGF-β1 - inhibited the CCl_4_-induced activation of the Hh signaling pathway - ↓ Shh, Ptch1, Smo, Gli1	[36]
		LX-2 and T6 cells BALB/c mice	- attenuated HSC activation - ↓ the mRNA levels of α-SMA and Col-1 in LX-2 and T6 cells - ↓ the expression of LncRNA-ROR in vitro - inhibited HSC proliferation via LncRNA-ROR - inhibited HSC activation via LncRNA-ROR-mediated NF-κB signaling - attenuated primary HSC activation and down-regulated LncRNA-ROR mRNA expression - ↓ α-SMA - ↓ the expression of LncRNA-ROR via miR-6499-3p - ameliorated liver function, attenuated fibrosis severity, inhibited HSC activation, and regulated LncRNA-ROR and NF-κB signaling in CCl_4_-induced experimental mice - ↓ serum ALT, AST, TBIL - ↓ α-SMA and Col-1 - ↓ the phosphorylation of NF-κB p65 - ↓ the phosphorylation levels of NF-κB p65, IκBα, and IKKα - ↓ IL-1β, IL-6, TNF-α, TGF-β1, COX-2 - inhibited the mRNA level of α-SMA and LncRNA-ROR in liver tissues	[37]
		LX-2 cells	- promotes FGF19 secretion by LX-2 cells - inhibited LPS-induced HSC proliferation and activation - ↓ α-SMA and Col1A1 - ↓ polymerization of actin F filaments in LX-2 cells - restored LPS-induced decrease in FGF19 and FGFR4 expression levels	[38]
		HSC-T6 and LX-2 cells	- ameliorated histopathological characteristics and hepatic fibrosis markers in mice - ↓ α-SMA, Col-1, TGF-β1 - inhibited activation of MAPK and P-Smad2/3L and P-Smad2C - ↑ phosphorylation of P-Smad3C - modulated MAPK pathway activation and Smad2/3 phosphorylation in TGF-β1-stimulated HSCs - ↓ P-ERK1/2, P-JNK1/2, P-p38, P-Smad2C, P-Smad2L, P-Smad3C, and P-Smad3L - ↑ P-Smad3C - inhibited the expression of PAI in TGF-β1-stimulated HSCs	[39]
	Sinapic acid	Sprague-Dawley rats	- prevented DMN-induced loss of body weight - ↓ AST, ALT - ↓ hepatic HYP content - ↓ MDA - ↓ TGF-β1, Col-1, α-SMA - ↓ NF-κB p65	[40]
Hydroxybenzoic acids	Gallic acid and dodecyl gallate	Wistar albino rats	- prevented the increase in relative liver weight and levels of triglycerides - restored serum hepatic enzyme activities - ↓ TBIL, ALT, AST, γ-GT - ↓ TBARS levels - ↑ GSH - ↓ lipid peroxidation levels - ↑ catalase, GPx, GR, GST - improved histopathologic alterations - ↑ the expression of p53 gene	[41]
	Protocatechuic acid	HSC-T6 cells C57BL/6 mice	- regulated cell viability in TNF-α-induced HSC-T6 cells via regulation on TGF-β signaling pathway - ↓ TGF-β, p-Smad2, p-ERK, and c-Jun - attenuated the alteration of phenotype associated with TAA-induced liver damage and fibrosis in mice - ↓ collagen - attenuated TAA-induced liver damage and fibrosis in mice - played a protective role in liver fibrosis through regulation of the TGF-β signaling pathway - ↓ the protein expression of p-Smad2, p-ERK, and c-Jun - inhibited the mRNA level of IL-6 and TNF-α in TAA-induced mice	[42]
	Vanillic acid	Sprague-Dawley rats HSC-T6 cells	- attenuated CCl_4_-induced liver fibrosis - ameliorated adipose degeneration of hepatocytes - ↓ infiltration of inflammatory cells - inhibited the MIF/CD74 signaling pathway in vivo - ↓ the mRNA and protein levels of MIF and CD74 - suppressed autophagy and activity of HSCs in vivo - ↓ α-SMA and LC3B - inhibited the MIF/CD74 signaling pathway and autophagy of HSCs in vitro - ↓ MIF, CD74, α-SMA, LC3B, and Col-1 - suppressed the proliferation and the migration of HSCs	[43]

Legend: ↑ increased/up-regulated; ↓ decreased/down-regulated; Akt, protein kinase B; ALB, albumin; ALP, alkaline phosphatase; ALT, alanine aminotransferase; AMPK, adenosine monophosphate-activated protein kinase; AST, aspartate aminotransferase; Atg5, autophagy-related gene 5; BAMBI, “bone morphogenetic protein” activin membrane-bound inhibitor; Bax, Bcl-2-associated X protein; Bcl-2, B-cell lymphoma-2; CAT, catalase; Ccl2, chemokine (C-C motif) ligand 2; CCl_4_, carbon tetrachloride; CIV, type IV collagen; Col-1, collagen 1; Col1α1, collagen type 1 alpha 1; Col1α2, collagen type 1 alpha 2; Col3α1, collagen type 3 alpha 1; Col4α1, collagen type 4 alpha 1; COX-2, cyclooxygenase-2; CTGF, connective tissue growth factor; DMN, dimethylnitrosamine; Ecm1, extracellular matrix protein 1; ERK, extracellular signal-regulated protein kinase; ERK1/2, extracellular signal-regulated kinases 1/2; FGF19, fibroblast growth factor; FGFR4, fibroblast growth factor receptor 4; GLB, globulin; Gli1, transcription factor glioma-associated oncogene homolog 1; GPx, glutathione peroxidase; GR, glutathione reductase; GSH, glutathione; GST, glutathione-S-transferase; HA, hyaluronic acid; HSCs, hepatic stellate cells; HYP, hydroxyproline; IL-1β, interleukin-1β; IL-6, interleukin-6; iNOS, inducible nitric oxide synthase; JNK, c-Jun N-terminal kinase; LC3B, microtubule-associated protein 2 light chain 3 type B; LN, laminin; LncRNA, long non-coding RNA; LOX, lysyloxidase; LPS, lipopolysaccharide; LX-2, human hepatic stellate cell line; MAPK, mitogen-activated protein kinase; MCP-1, monocyte chemoattractant protein-1; MDA, malondialdehyde; MIF, macrophage migration inhibitory factor; MMP-9, matrix metalloproteinase 9; MPHs, mouse primary hepatocytes; mTOR, mammalian target of rapamycin; MYD88, myeloid differentiation primary response 88; NF-κB, nuclear factor kappa B; NLRP3, NLR family pyrin domain containing 3; NOX2, nicotinamide adenine dinucleotide phosphate oxidase-2; Nrf2, nuclear factor erythroid 2-related factor 2; PAI, plasminogen activator inhibitor; p-Akt, phosphorylated protein kinase B; PCIII, procollagen type III; PDGFRβ, platelet-derived growth factor receptor beta; PDGF-βR, platelet-derived growth factor receptor beta; p-ERK, phosphorylated extracellular signal-regulated protein kinase; PI3K, phosphatidylinositol 3-kinase; PIIIP, procollagen III peptide; p-JNK, phosphorylated c-Jun N-terminal kinase; p-mTOR, phosphorylated mammalian target of rapamycin; p-Smad2, phosphorylated Smad2; P-Smad2/3L, phosphorylation of Smad2/3 at linker regions; P-Smad2C, phosphorylation of Smad2 at C-terminal linker regions; p-Smad3, phosphorylated Smad3; P-Smad3C, phosphorylation of Smad3 at C-terminal linker regions; Ptch1, membrane protein receptor protein patched homolog 1; PTP1B, protein tyrosine phosphatase 1B; ROR, regulator of reprogramming; ROS, reactive oxygen species; Shh, Sonic hedgehog protein; Smo, membrane protein receptor Smoothened; SOD, superoxide dismutase; TAA, thioacetamide; TBIL, total bilirubin; TGF-β1, transforming growth factor beta 1; TIMP-1, tissue inhibitor of metalloproteinases 1; TLR4, toll-like receptor 4; TNF-α, tumor necrosis factor alpha; α-SMA, alpha smooth muscle actin; γ-GT, γ-glutamyl transpeptidase.

### 2.2. Flavonoids

Flavonoids are a class of plant pigments increasingly utilized in drug development and nutraceutical applications [44]. These natural phenolic compounds feature a phenyl benzo (γ) pyrone-derived structure, comprising two benzene rings (A and B) connected by a pyrane ring (C) [45].

The effectiveness of orally administered flavonoids is limited due to their low dissolution rate, partial degradation in the acidic gastric environment, reduced permeability, and extensive first-pass metabolism before reaching systemic circulation [46].

Flavonoids are categorized into different subclasses based on the specific carbon atom of ring C to which ring B is attached and the degree of unsaturation and oxidation of ring C. These subclasses include flavan-3-ols (also known as flavanols or catechins), flavonols, flavones, flavanones, isoflavones, anthocyanidins, and chalcones [47,48].

#### 2.2.1. Flavanols

Flavanols (IUPAC name: 3-hydroxy-2-phenylchromen-4-one) are a subclass of flavonoids and serve as secondary metabolites in plants [49]. Their chemical structure includes a hydroxyl group (-OH) on the third carbon atom (C3) and a carbonyl group (C=O) on the fourth carbon atom of the central heterocyclic ring [50].

Table 2 summarizes the pharmacological effects of several flavanol compounds on liver fibrosis, highlighting their ability to reduce fibrosis, oxidative stress, inflammation, and hepatic stellate cell activation. Each compound—epigallocatechin-3-gallate (EGCG), dihydromyricetin, hesperetin and its derivatives, hesperidin, liquiritigenin, naringenin, and naringin—demonstrates protective effects against liver damage through various mechanisms, including the modulation of signaling pathways like TGF-β1/Smad, PI3K/Akt, and cGAS-STING, ultimately contributing to the attenuation of liver fibrosis and improvement in liver function.

#### 2.2.2. Flavonols

Flavonols are bioavailable compounds with multiple therapeutic benefits, such as hepatoprotective activity, free radical scavenging, cardioprotective, antiviral, antibacterial, and antineoplastic properties [73]. Flavonols contain a central structure of 3-hydroxyflavones, also known as 3-hydroxy-2-phenylchromen-4-one [74,75]. Flavonols are distinguished from other groups of flavonoids by the hydroxylation of one of the benzene rings. Each flavonol presents a distinct pattern of hydroxylation of the benzene ring [76,77]. The free forms of flavonols are called aglycones. The latter have a common structure of a 3-hydroxyflavone backbone and are distinguished by the position of the hydroxyl groups. The number of hydroxyl groups significantly contributes to the bioactivity of these compounds [78,79].

Table 3 summarizes the pharmacological effects of various flavonols in reducing liver fibrosis. These compounds—fisetin, galangin, isorhamnetin, kaempferol, dihydrokaempferol, morin, myricetin, myricitrin, and quercetin—work primarily by inhibiting key fibrotic pathways, including PI3K/Akt, Wnt/β-catenin, TGF-β1-Smad, and ERK1/2 signaling pathways, by reducing inflammation and oxidative stress, suppressing hepatic stellate cell activation, and enhancing antioxidant defenses.

#### 2.2.3. Flavones

Flavones constitute another subclass of flavonoids. Their core structure includes a double bond between the C2 and C3 positions and a ketone group at the C4 position on ring C. The molecular formula for flavones is in [20,105,106]. Typically, flavones have a hydroxyl group at the fifth position of ring A, with additional hydroxylation potentially occurring at other positions, such as the seventh position of ring A or the 3′ and 4′ positions of ring B [107].

Table 4 provides a detailed summary of the pharmacological effects of flavones in combating liver fibrosis. These flavones act by reducing oxidative stress, inflammation, and collagen deposition, primarily through the inhibition of key fibrogenic signaling pathways like TGF-β/Smad and PI3K/Akt (baicalein, chrysin, isovitexin). They also suppress the activation and proliferation of hepatic stellate cells (HSCs), promote apoptosis and autophagy, and restore the balance of extracellular matrix (ECM) components (diosmin, isoorientin, ligustroflavone, isovitexin). Additionally, some flavones enhance antioxidant defenses and modulate pathways such as Nrf2 (alpinetin), cGAS-STING (oroxylin A), and Hippo/YAP and autophagy pathways (nobiletin), repressing the miR-17-5p/Wnt/β-catenin signaling (diosmin), suppressing TGF-β1-induced Smad and AKT signaling (luteolin), inhibiting the TLR2/TLR4 pathway (luteoloside), blocking the p38 MAPK and PDGF-Rβ signaling pathways (tricin), and contributing to the attenuation of liver fibrosis and the restoration of normal liver structure and function in various cell and animal models.

#### 2.2.4. Flavanones

Flavanones are a subclass of flavonoids characterized by their distinct chemical structure. They have a flavan nucleus with a saturated three-carbon chain and a hydroxyl group attached to the second carbon (C2). This structure differentiates them from other flavonoids, such as flavones and flavonols, which have a double bond between C2 and C3. Flavanones are commonly found in citrus fruits such as oranges, lemons, and grapefruits, and they contribute to the characteristic bitterness of these fruits. Examples of flavanones include naringenin, hesperetin, and eriodictyol.

Flavanones have been studied for their potential health benefits, including antioxidant, anti-inflammatory, and anticancer properties. Their bioavailability and metabolism are subjects of ongoing research to better understand their therapeutic potential.

Table 5 highlights how flavanones mitigate liver fibrosis by inhibiting key fibrogenic pathways, via the SIRT1/TGF-β1/Smad3 signaling pathway, enhancing autophagy, and inhibiting the PI3K/AKT/mTOR pathway (ampelopsin), by reversing activated HSCs to their quiescent state, modulating autophagy, and inhibiting the mTOR signaling pathway (naringin), or by reducing oxidative stress and inflammation, enhancing antioxidant defenses via the Nrf2/HO-1 pathway, and inhibiting HSC activation through the TGF-β/Smad and PI3K/Akt signaling pathways (pinocembrin), leading to reduced fibrosis and improved liver function.

#### 2.2.5. Isoflavones

Isoflavones are a subclass of flavonoids, which are naturally occurring compounds found in plants. Unlike other flavonoids, isoflavones have a unique structural feature where the B ring is attached to the C3 position of the central C ring, rather than the C2 position. This structural variation imparts distinct biological activities to isoflavones.

Isoflavones are predominantly found in legumes, with soybeans being the most significant source. Some of the well-known isoflavones include genistein, daidzein, and glycitein.

Table 6 highlights how isoflavones mitigate liver fibrosis by reducing oxidative stress, inflammation, and collagen deposition, while inhibiting key fibrogenic pathways such as JAK2/STAT3, TGF-β/Smad, and ERK1/2 (calycosin, genistein, puerarin, glabridin, soy isoflavone, tectorigenin). These compounds also promote the balance of matrix metalloproteinases (MMPs) and tissue inhibitors (TIMPs), enhance antioxidant defenses, and modulate HSC activation, contributing to the attenuation of fibrosis and improvement in liver function.

#### 2.2.6. Anthocyanidins

Anthocyanidins are pigments responsible for the colors in plants, flowers, and fruits [48]. Anthocyanins from blueberries have been shown to regulate the epigenetic modifications of hepatic stellate cells (HSCs), thereby intervening in the treatment of hepatic fibrosis [154,155,156].

Table 7 highlights how anthocyanidins reduce liver fibrosis by inhibiting key fibrotic processes such as HSC activation, inflammation, oxidative stress, and collagen deposition, while enhancing antioxidant defenses, promoting apoptosis, and modulating signaling pathways such as Nrf2 and TGF-β/Smad, contributing to improved liver function and reduced fibrosis.

#### 2.2.7. Chalcones

Chalcones are a subclass of flavonoids characterized by their unique open-chain structure. Unlike other flavonoids that have a closed ring system, chalcones consist of two aromatic rings (A and B) joined by a three-carbon α,β-unsaturated carbonyl system.

Chalcones are found in various plant species and are known for their bright yellow pigments. They serve as precursors in the biosynthesis of other flavonoids and isoflavonoids through the chalcone isomerase-catalyzed cyclization.

Table 8 demonstrates that chalcones effectively combat liver fibrosis by targeting several critical mechanisms. These compounds inhibit the activation of hepatic stellate cells (HSCs), reduce oxidative stress, and suppress inflammatory responses. Additionally, chalcones promote apoptosis in fibrotic cells, decrease collagen deposition, and modulate key signaling pathways such as Nrf2/HO-1, NF-κB, and metabolic processes like glycolysis. Together, these actions lead to significant reductions in fibrosis and improvements in liver function across various experimental models.

### 2.3. Stilbenes

Stilbenes are organic compounds characterized by a 1,2-diphenylethylene structure, commonly found in plants such as grapes, berries, and peanuts. Known for their potent antioxidant properties, these phytochemicals have been extensively researched for their potential health benefits. Resveratrol, a notable stilbene, offers liver protection against damage from chemicals, cholestasis, and alcohol; improves glucose metabolism and lipid profiles; and reduces liver fibrosis and steatosis [173]. It regulates fibrogenesis by reducing portal pressure, inhibiting hepatic stellate cell activation, and enhancing endothelial function, as well as modulating key signaling pathways like NF-κB and PI3K/Akt [174,175], and inducing autophagy via the microRNA-20a/PTEN/PI3K/AKT axis [176].

In liver fibrosis models, such as those induced by dimethylnitrosamine (DMN), resveratrol reduces inflammatory cell infiltration and fibrosis, lowering MDA levels, increasing GPx and SOD levels, and inhibiting inflammatory mediators like NO, TNF-α, and IL-1β [177,178,179]. Additionally, resveratrol alleviates mercury-induced liver fibrosis by activating the Sirt1/PGC-1α pathway and regulating the microbiota–gut–liver axis, enhancing the abundance of Bifidobacterium [180]. In obstructive jaundice, it protects liver function by modulating lipid metabolism, reducing oxidative stress, and down-regulating targets like mTOR and CYP enzymes [181]. Piceatannol, a resveratrol analog, effectively protects against CCl4-induced liver fibrosis in mice by improving liver function, reducing collagen deposition, and suppressing fibrosis markers via the TGF-β/Smad pathway. It also alleviates oxidative damage, highlighting its potential as a preventive agent for liver fibrosis [182].

Pterostilbene, found in grapes and berries, inhibits DMN-induced liver fibrosis in rats by improving liver function, reducing fibrotic changes, and decreasing hepatic stellate cell activation. It lowers serum ALT and AST levels, improves histopathology, and reduces fibrosis markers like α-SMA, TGF-β1, and MMP2, likely through the inhibition of the TGF-β1/Smad signaling pathway [183]. In another study, pterostilbene with a superior pharmacokinetic profile showed stronger anti-fibrotic effects than hydroxylated stilbenes in a CCl_4_-induced rat liver fibrosis model. It significantly reduced fibrosis markers and down-regulated key signaling pathways, demonstrating more potent protective activity.

Another stilbene, mulberroside A, reduces CCl_4_-induced liver fibrosis in mice by inhibiting the pro-inflammatory response and cytokine expression, providing significant liver protection without directly affecting hepatic stellate cell proliferation [184].

## 3. Polyphenol-Based Drug Delivery Systems and Hepatic Fibrosis

Nanoparticulate drug delivery systems significantly enhance the pharmacokinetics of drugs, including their absorption, metabolism, and excretion [185,186,187]. Compared to traditional formulations, nanoencapsulation offers several advantages, such as improved solubility and bioavailability, targeted drug delivery, consistent drug release, reduced dosage, and fewer side effects [188].

Nanoparticles (NPs) play a crucial role in improving the efficiency of co-delivery methods due to their ability to easily cross cell membranes because of their small size, enhance drug kinetics, and escape lysosomal degradation following endocytosis [189,190,191]. The main challenge of co-delivery systems is using carriers to simultaneously transport drugs with different properties [192]. Various modified carriers, such as liposomes, micelles, and polymeric NPs, have been employed to enhance the efficiency of co-delivery systems [193].

Polymeric nanoparticles, solid lipid nanoparticles, polymeric micelles, dendrimers, liposomes, nanocapsules, nanogels, nano-emulsions, and carbon nanotubes are examples of novel drug delivery systems (NDDSs). These systems, composed of biocompatible and biodegradable materials, offer numerous advantages over conventional dosage forms, such as controlled drug release, improved stability, and reduced adverse effects [194,195].

Nanocarriers ensure site-specific delivery of therapeutics, thereby improving bioavailability, stability, solubility, controlled release of active ingredients, and prolonged drug action [196,197]. Additionally, nanocarriers protect drugs from metabolic degradation [198]. Examples of nanocarriers include nanocapsules, nanospheres, nano-emulsions, and nano-sized vesicular carriers [196].

Polymeric NPs are effective carriers for the oral administration of flavonoids. They enhance physicochemical stability, increase solubilization and bioavailability by improving absorption at the enterocyte level, and maintain therapeutic levels in blood and plasma with a significant increase in mean residence time [199,200]. Polymeric NPs protect against degradation, provide controlled release of therapeutic agents, and enhance specific transport [201].

Poly(D,L-lactic-co-glycolic acid) (PLGA) is a biopolymer used in the preparation of NPs for various therapeutic applications. It protects compounds from degradation and offers sustained drug release [202,203]. The US Food and Drug Administration (FDA) has approved PLGA due to its properties, including biocompatibility, biodistribution, and biodegradability [204,205]. PLGA has been used in numerous drug delivery systems, both targeted and non-targeted [206,207].

PLGA nanoparticles are taken up by endocytosis, releasing the drug in intracellular locations, thereby improving therapeutic action and reducing side effects [207,208]. The rapid absorption of PLGA nanoparticles by the reticuloendothelial system can be significantly reduced by modifying their surface with poly(ethylene glycol) (PEG). This modification extends the circulation time of nanosystems in the blood, allows targeting to tissues, and prevents opsonization [209,210]. These nanoparticles with hydrophilic surfaces have shown improved permeability, presumably due to the prolonged residence time of the carrier in the blood [211,212]. The surface modification of nanoparticles with polyethylene glycol (PEG) extends circulation time, reduces non-specific interactions, and favors accumulation in tumors due to increased permeability and retention [212,213].

Polycaprolactone (PCL), a biodegradable polymer, is suitable for controlled drug release due to its high permeability to many drugs and lack of toxicity [214]. PCL can also form blends with other polymers [215].

Table 9 highlights the effectiveness of polyphenol-based drug delivery systems in combating liver fibrosis by leveraging advanced formulations that enhance the bioavailability, targeting, and therapeutic efficacy of active compounds. These formulations, including nanoparticles, nanocomplexes, liposomes, and exosomes, significantly improve the delivery of polyphenols like galangin, quercetin, chrysin, luteolin, hesperidin, naringenin, silibinin, silymarin, and curcumin. The enhanced delivery systems allow these compounds to more effectively reduce oxidative stress, inflammation, and fibrotic markers such as ALT, AST, TGF-β1, and collagen deposition. Additionally, these systems improve the modulation of key signaling pathways like TGF-β/Smad, NF-κB, and Nrf2, leading to a better preservation of liver architecture and function in various experimental models of liver fibrosis.

## 4. Conclusions and Perspectives

**Figure 1:**
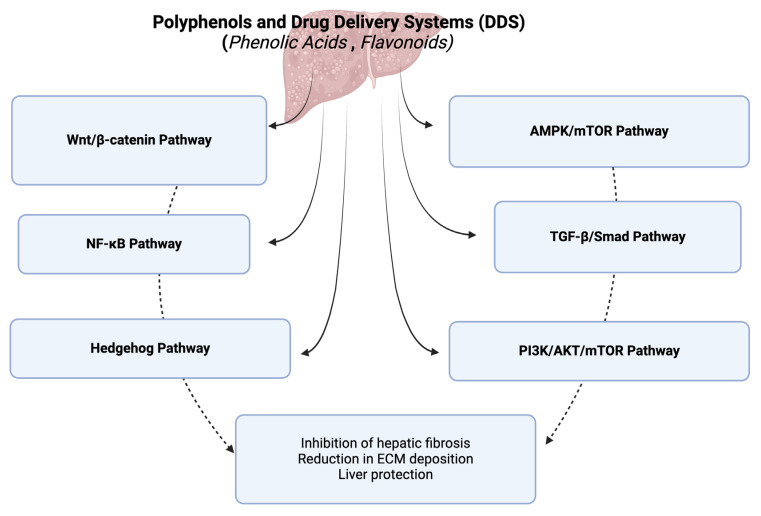
A diagram of signaling pathways modulated by polyphenols and their drug delivery systems in hepatic fibrosis. This figure was created with BioRender.com.

The integration of plant-based therapeutics with advanced nanoparticulate drug delivery systems offers a promising approach to treating hepatic fibrosis. Polyphenols, phenolic acids, and flavonoids have shown significant potential in mitigating hepatic fibrosis by targeting multiple signaling pathways involved in the disease’s progression. These natural compounds exert anti-fibrotic effects through mechanisms such as the inhibition of hepatic stellate cell activation, reduction in extracellular matrix deposition, and modulation of inflammatory responses. Overall, the signaling pathways modulated by these natural compounds in the context of hepatic fibrosis, which lead to the inhibition of hepatic fibrosis, reduction in extracellular matrix (ECM) deposition, and overall liver protection, are the TGF-β/Smad pathway, AMPK/mTOR pathway, Wnt/β-catenin pathway, NF-κB pathway, PI3K/AKT/mTOR pathway, and hedgehog pathway (Figure 1).

**Figure 2:**
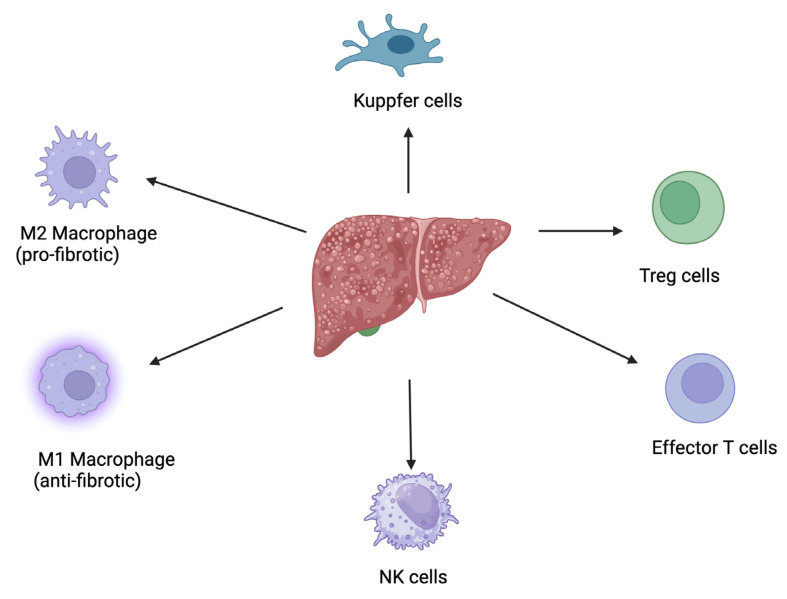
A diagram illustrating the role of immune cells and immune responses mediated by polyphenols in the resolution of liver fibrosis. This figure was created with BioRender.com.

However, the modulation of immune cells and immune responses mediated by polyphenols (Figure 2) are key events in the resolution of liver fibrosis: (a). the modulation of macrophage polarization from the pro-fibrotic M2 phenotype to the anti-fibrotic M1 phenotype, which subsequently produces pro-inflammatory cytokines that help clear apoptotic cells and degrade the extracellular matrix; (b). inhibition of hepatic stellate cell activation, through reducing the secretion of pro-fibrotic cytokines like TGF-β from immune cells; (c). regulation of T-cell responses, particularly influencing the balance between regulatory T-cells (Tregs) and effector T-cells; (d). reduction in oxidative stress and inflammation, by scavenging reactive oxygen species (ROS) and inhibiting the NF-κB signaling pathway, which are key contributors to liver fibrosis; (e). enhancement in autophagy in immune cells and hepatic stellate cells, a process that facilitates the clearance of damaged cells and reduces the deposition of extracellular matrix proteins; (f). modulation of the Kupffer cell activity, by reducing their pro-inflammatory cytokine production and promoting their role in clearing fibrotic tissue; (g). enhancing the cytotoxic activity of NK cells against activated hepatic stellate cells, leading to their apoptosis and reducing fibrogenesis.

Nanoparticulate drug delivery systems further enhance the therapeutic efficacy of these bioactive compounds by improving their solubility, bioavailability, and pharmacokinetics. Polymeric nanoparticles, solid lipid nanoparticles, polymeric micelles, and other novel drug delivery systems have demonstrated the ability to deliver therapeutic agents with high precision and reduced side effects.

Future research should focus on translating these findings into clinical applications. Rigorous clinical trials are needed to validate the safety and efficacy of plant-based therapeutics and nanoparticulate drug delivery systems in human subjects.

In conclusion, the convergence of plant-based therapeutics and innovative drug delivery systems represents a promising frontier in the fight against hepatic fibrosis. Continued research and development in this area hold the potential to significantly improve the management and treatment of this debilitating condition.

## Figures and Tables

**Table 2:** Pharmacological effects of flavanols in liver fibrosis.

Bioactive Compounds	Cell Lines/ Animal Model	Pharmacological Effects	Reference
Epigallocatechin-3-gallate (EGCG)	Wistar rats	- AST and AST serum levels - prevented deposition of collagen in the hepatic parenchyma - ↓ total collagen content - ↓ oxidative stress and lipid peroxidation - ↓ MDA levels in serum - ↓ HA and collagen type IV in serum - ↓ serum OPN levels - inhibited activation of HSC cells - ↓ the expression of α-SMA - ↓ production of 4-HNE - inhibited protein expression of OPN - ↓ mRNA levels of α-SMA, OPN, Col-1, and Col-3	[51]
	HepG2 cells Sprague-Dawley rats	- ↑ the expression of miRNAs 221, 181a, and 10b in vitro - miRNAs 221, 181a, and 10b ↓ OPN mRNA when administered alone - EGCG required the expression of miR-221 to down-regulate OPN protein and its associated fibrogenic properties - in vivo, EGCG prevented toxin-induced fibrosis - ↓ OPN expression - ↑ miR-221, miR-181a, and miR-10b	[52]
	Human hepatic stellate LX-2 cells Sprague-Dawley rats	- ameliorated liver necrosis, inflammation, and fibrosis - suppressed the gene expression associated with liver inflammation and fibrogenesis: TNF-α, IL-1β, TGF-β1, MMP-9, α-SMA, and Col1α1 - suppressed TGF-β1-stimulated expression of Col1α1, MMP-2, MMP-9, TGF-β1, TIMP-1, and α-SMA in LX-2 cells - suppressed the phosphorylation of Smad2/3 and Akt in the livers of bile duct ligation (BDL) rats and in TGF-β1-stimulated LX-2 cells	[53]
	C57BL/b6 mice	- ↓ CCl_4_-induced necrosis, cellular injury, and formation of nitrotyrosine - serum ALT - ↓ CCl_4_-induced collagen accumulation in the liver - ↓ the expression levels of pro-inflammatory mediators: iNOS, TNF-α, and COX-2 - attenuated CCl_4_-modified activity of NF-κB - ↓ the mRNA expression levels of α-SMA, TGF-β1, pro-collagen-I, MMP-2, and TIMP-1	[54]
	Wistar rats	- ↓ serum levels of AST and ALT - attenuated hepatic fibrosis - ↓ HYP in the liver - ↓ the expression of PDGFRβ and IGF-1R mRNAs - inhibited HSC activation - ↓ the expression of PDGFRβ and α-SMA	[55]
	Sprague-Dawley rats Rat HSCs	- ameliorated liver injury - ↓ AST and ALT - ↓ liver fibrosis and HSC activation in CCl_4_-treated rats - ↓ the fibrotic areas - ↓ HYP level - ↓ α-SMA - ↑ hepatic GSH levels - ↓ the hepatic level of TBARS - ↓ MMP-2 - inhibited pro-MMP-2 activation in vitro - ↓ concanavalin A induced MMP-2 activation through direct inhibition of MT1-MMP activity - ↓ mRNA expression and protein level of MMP-2	[56]
Dihydromyricetin	ICR mice	- alleviated TAA-induced chronic hepatic injury - ↓ the levels of ALT and AST in serum - improved TAA-induced liver histopathological damage - ↓ collagen deposition - alleviated TAA-induced hepatotoxicity - ↑ GSH and SOD - ↓ MDA - ↓ TGF-β1 and α-SMA - attenuated liver fibrosis by inhibiting TAA-induced inflammatory injury - ↓ NF-κB - inhibited the expression of pro-inflammatory factors TNF-α and IL-1β - inhibited hepatocyte apoptosis by regulating the PI3K/Akt signaling pathway - ↑ p-PI3K and p-AKT - ↓ Bax, cleaved caspase-9, and cleaved caspase-3 - ↑ Bcl-2 and Bcl-XL	[57]
	LX2 and NK92 cells C57BL/6 J mice	- ameliorated CCl_4_-induced liver fibrosis and HSC activation in vivo - ↓ the serum levels of ALT and AST - ↓ the expressions of Col1α1, Col1α2, TIMP-1, α-SMA, and desmin - ↑ MMP-1 - inhibited TGF-β1-induced HSC activation in vitro - suppressed the TGF-β1-induced increase in the viability of LX2 cells - ↓ the expression of Col-1 and α-SMA proteins in TGF-β1-treated LX2 cells - inhibited HSC activation by inducing autophagy - ↑ the expression of MAP1LC3B2 (LC3B-II) in the liver from CCl_4_-treated mice - ↓ SQSTM1 - ↑ Beclin1 and Atg3 - promoted the activation of hepatic NK cells in mice - ↑ the frequency of hepatic NK cells - ↑ the frequency of cells expressing NKG2D - ↑ the frequency of IFN-γ expression - enhanced NK cell-mediated killing of HSCs via IFN-γ in vitro - suppressed the TGF-β1-induced decrease in NK92 cell viability - ↑ the percentage of apoptotic and necrotic cells - induced IFN-γ production in NK92 cells through the AhR-NF-κB/STAT3 signaling pathway - ↑ mRNA expression levels of AhR and CYP1A1 - ↑ P-STAT3/STAT3 ratio	[58]
Hesperetin	C57BL/6J mice HSC-T6 cells	- alleviated hepatic injury and fibrosis - ↓ the serum levels of ALT and AST - ↓ the gene and serum levels of IL-6 and TNF-α - ↓ histological scores - ↓ collagen deposition - suppressed the formation of ECM - suppressed the serum level of LN and HA - ↓ HYP - inhibited the liver fibrosis through down-regulation of the TGF-β1/Smad pathway - ↓ the protein levels of TGF-β1 - ↓ the phosphorylation of Smad2 and Smad3 - inhibited the activation and proliferation of HSC-T6 cells, and promoted apoptosis - ↓ caspase 3 and Bax - ↑ Bcl-2 - inhibited the protein level of α-SMA and Col-1	[59]
Hesperetin derivative	LX-2 cells C57BL/6J mice	- exhibited hepatoprotective and anti-fibrotic effect in vivo - ↓ serum levels of ALT, AST, and ALP - inhibited collagen deposition - ↓ serum levels of TGF-β1 and HA - ↓ HYP in liver tissues - inhibited the up-regulation of liver fibrogenesis markers α-SMA, Col1α1, Col3α1, and TIMP-1 in primary HSCs and suppressed inflammatory responses in primary liver macrophages from hepatic fibrosis mice - protected against CCl_4_-induced inflammatory responses in vivo - ↓ F4/80^+^ macrophage infiltration - ↓ mRNA levels of inflammatory genes (MCP-1, TNF-α, IL-1β, and IL-6) - ↓ the serum levels of TNF-α and IL-1β - suppressed TNF-α and IL-1β protein expression - inhibited the phosphorylation and activation of NF-κB-P65 - suppressed the protein expression of α-SMA and Col1α1 and inhibited mRNA levels of α-SMA, Col1α1, Col3α1, TIMP-1, and PAI-1 in TGF-β1-activated LX-2 cells - attenuated CCl_4_-induced mouse liver fibrogenesis and TGF-β1-induced fibrotic responses in LX-2 cells via Gli-1-dependent mechanisms - inhibited mRNA levels and protein expression of Gli-1 and Shh - ↓ expression of α-SMA and Gli-1 in TGF-β1-activated LX-2 cells - promoted apoptosis response in TGF-β1-activated LX-2 cells - ↑ the level of Bax and cleaved-caspase3	[60]
Hesperetin derivative	C57BL/6J mice HSC-T6 cells	- exhibited hepatoprotective and anti-fibrotic effect in vivo - ↓ the serum level of AST and ALT - ↓ mRNA and the protein levels of Col1α1 and α-SMA - higher concentrations of HD exerted anti-fibrotic effect in TGF-β1-stimulated HSC-T6 cells in vitro - ↑ Ptch1 expression in TGF-β1-stimulated HSC-T6 cells and CCl_4_-induced mice - ↓ the activation of HSCs by targeting Ptch1 - ↓ TGF-β1-activated HSC-T6 cell proliferation - induced cell cycle arrest in the S phase by reducing the ratio of the G2/M phase in HSC-T6 cells stimulated with TGF-β1	[61]
Hesperetin derivative-7	KunMing mice HSC-T6 cells	- attenuates liver fibrosis - ↓ the adipose degeneration of hepatocytes - ↓ the immigration of inflammatory cells - ↓ collagen fibers - ↓ the mRNA and protein levels of α-SMA and Col1α1 - ↓ the viability of PDGF-BB-induced HSC-T6 cells - inhibited the proliferation and the activation of PDGF-BB-treated HSC-T6 cells - inhibited the activation and proliferation of PDGF-BB-induced HSC-T6 cells by targeting Wnt/β-catenin signaling pathway - ↓ the mRNA and protein levels of β-catenin, c-myc, and cyclin D 1	[62]
Hesperetin derivative (HD-11)	Sprague-Dawley rats HSC-T6 cells	- exhibited hepatoprotective effect in vivo - ↓ the protein levels of α-SMA - ↓ the levels of ALT and AST - alleviated ECM deposition in rats with liver fibrosis - ↓ the expression of α-SMA and Col1α1 in TGF-β1-induced HSC-T6 cells - promoted the expression of PTEN in vivo and in vitro - inhibited activated HSC-T6 cell proliferation induced by TGF-β1 via promoting cell cycle arrest - suppressed cell proliferation through PTEN/AKT pathway in TGF-β1-induced HSC-T6 cells - inhibited the expression of p-AKT in vivo and in vitro	[63]
Hesperetin derivative-16 (HD-16)	LX-2 cells C57BL/6J mice	- alleviated CCl_4_-induced liver injury and fibrosis in mice - ↓ the collagen deposition - ↓ mRNA and protein levels of fibrogenic α-SMA and Col1α1 - protected against CCl_4_-induced inflammatory responses in vivo - suppressed the levels of TNF-α and IL-1β - promoted the levels of IL-10 and IL-13 in serum and liver tissue homogenates - attenuated activation of HSCs in vitro - inhibited the viability of LX-2 cells - suppressed the protein expression of α-SMA and Col1α1 in TGF-β1-activated LX-2 cells - ↓ the mRNA levels of α-SMA, Col1α1, Col3α1, and TIMP-1 - suppressed the release of pro-inflammatory factors TNF-α and IL-1β - facilitated the release of anti-inflammatory factors IL-10 and IL-13 in TGF-β1-activated LX-2 cells - promoted SIRT3 expression and activity in vivo and in vitro - ↑ the mRNA level of SIRT3, but not SIRT1 - ↑ SIRT3 deacetylase activity - ↑ SIRT3 protein expression - SIRT3 depletion attenuated the anti-fibrotic effects of HD-16 - ↑ the expression of SIRT3 via activation of AMPK/SIRT3 pathway - attenuated inflammation and fibrosis through AMPK/SIRT3 pathway in TGF-β1-activated LX-2 cells	[64]
Hesperidin	Wistar rats	- improved the histological morphology and structure of the liver parenchyma - ↓ the levels of liver enzymes (AST, ALT, ALP, LDH) and total and direct bilirubin - ↓ NO, MDA, PC - ↓ inflammatory gene expression: TGF-β1, iNOS, caspase-3, and α-SMA - ↑ the levels of total antioxidant capacity and GSH, SOD, and CAT enzyme activity	[65]
	Wistar rats	- prevented serum markers of liver damage - ↓ ALT and γ-GT - exhibited antioxidant activity - ↓ MDA - ↑ GSH, the GSH/GSSG ratio, and total glutathione - preserved glycogen content - ↓ collagen deposits - exhibited immunomodulatory activity - ↓ the protein expression of NF-κB p65, TGF-β, CTGF, and IL-1β	[66]
Liquiritigenin	C57BL/6J mice Primary HSCs	- ameliorated CCl_4_-induced liver fibrosis - ↓ collagen deposition and α-SMA level - presented an inhibitory role in HSC proliferation - suppressed HSC transdifferentiation - α-SMA mRNA - ↓ actin fibers in α-SMA protein - inhibited HSC activation - ↓ the mRNA expression level of Col1α1 - inhibited liver fibrosis via regulation of miR-181b and PTEN - ↑ PTEN in vivo and in vitro - ↓ miR-181b expression via Sp1	[67]
	C57BL/6 mice LX-2 cells	- protected the liver from CCl_4_-induced toxicity - ↓ ALT - ameliorated CCl_4_-induced liver fibrosis - ↓ the regions of hepatic degeneration and inflammatory cell infiltration - ↓ α-SMA - ↓ collagen fibers - ↓ HNE - suppressed TGF-β1/Smad signaling and HSC activation in vitro - ↓ α-SMA - suppressed the TGF-β1-induced gene expression of PAI-1 and MMP-2 - ↓ Smad3 phosphorylation - inhibited dysregulation of Smad4 and 7 induced by TGF-β1 - activated Hippo signaling - ↑ activation of LATS1 with the induction of YAP phosphorylation - ameliorated experimental liver fibrosis by acting on the TGF-β1/Smad and Hippo/Yap pathways	[68]
Naringenin	C57BL/6J mice Human LX2 and L02 cells	- ↓ liver fibrosis in mice - ↓ the levels of ALT, AST, ALP, ALT, LN, HA, PC-III, and IV-C - ↓ the expression of inflammatory factors, such as IL-1β, IL-6, IL-18, and TNF-α - targeted cGAS in HSCs - ↓ α-SMA and cGAS - inhibited the activation of HSCs in vitro - ↓ mRNA levels of α-SMA and α1-procollagen - ↓ α-SMA and α-SMA and Col-1 protein expression - ↓ the secretion of inflammatory factors in LX2 cells by inhibiting the cGAS-STING pathway - ↓ the expression of cGAS and STING mRNA - inhibited the mRNA levels of IL1-β, IL-6, IL-8, and NF-κB - ↓ the expression of IRF3 protein	[69]
	Wistar rats	- reversed liver damage, biochemical and oxidative stress marker elevation, and fibrosis - ↓ NF-κB, IL-6, IL-10, IL-1β - reversed collagen accumulation by modulating the synthesis and degradation of ECM - ↓ HYP and Col-1 - ↓ CTGF, MMP-2, MMP-9, MMP-13, and TIMP-1 protein levels - inhibited TGF-β pathway - ↓ TGF-β, α-SMA - ↑ Smad7 - inhibited the pro-fibrogenic JNK-Smad3 pathway - ↑ JNK activation and Smad3 phosphorylation	[70]
	Wistar rats	- prevented necrosis and cholestasis and improved liver biosynthetic capacity in CCl_4_-treated rats - ↓ ALT, AP, γ-GT - prevented the depletion of hepatic glycogen - prevented the oxidative stress caused by chronic liver damage - ↓ MDA levels - ↑ GSH level - ↑ GPx enzymatic activity - prevented inflammation and necrosis in CCl_4_-treated rats by maintaining normal NF-κB, IL-1, and IL-10 levels - ↓ collagen deposition - preserved the normal activity of MMP-9 and MMP-2 in experimental liver cirrhosis - blocked HSC transdifferentiation and Col-1 synthesis by inhibiting pro-fibrogenic proteins - ↓ TGF-β, α-SMA, CTGF, Col-1, and MMP-13 protein expression - ↑ Smad7 protein levels - inhibited HSC proliferation by blocking the JNK-pSmad3L pathway - prevented JNK activation, the elevation of Smad3 protein levels, and phosphorylation in the linker region	[71]
Naringin	Sprague-Dawley rats	- protected rat liver from TAA-induced hepatic injury and inflammatory necrosis - ↓ ALT, AST, TBIL - ↑ albumin and total protein - restored the normal liver architecture - suppressed TAA-induced oxidative stress in rat liver - ↓ MDA - ↑ CAT, GPx, SOD - modulated the cytokine expression - ↓ IL-6, IL-1β, IFN-γ - ↑ IL-10 - attenuated TAA-induced hepatic fibrogenesis - ↓ collagen deposition - ↓ α-SMA, TGF-β1, fibronectin - induced HSC apoptosis through disruption of p-Akt/Akt pathway - suppressed Akt phosphorylation - ↓ the ratio of p-Akt/Akt - ↑ caspase-3	[72]

Legend: ↑ increased/up-regulated; ↓ decreased/down-regulated; 4-HNE, 4-hydroxy-2-nonenal; AhR, aryl hydrocarbon receptor; Akt, protein kinase B; ALP, alkaline phosphatase; ALT, alanine aminotransferase; AMPK, AMP-activated protein kinase; AP, alkaline phosphatase; AST, aspartate aminotransferase; Atg3, autophagy-related gene 3; Bax, Bcl-2-associated X protein; Bcl-2, B-cell lymphoma-2; Bcl-XL, B-cell lymphoma-extra large; BDL, bile duct-ligated; CAT, catalase; CCl_4_, carbon tetrachloride; cGAS, cyclic guanosine monophosphate-adenosine monophosphate synthase; Col-1, collagen 1; Col1α1, collagen type I alpha 1; Col1α2, collagen type I alpha 2; Col-3, collagen 3; Col3α1, collagen type 3 alpha 1; CTGF, connective tissue growth factor; ECM, extracellular matrix; EGCG, epigallocatechin-3-gallate; eTIMP-1, tissue inhibitor of metalloproteinases 1; Gli-1, glioma-associated oncogene-1; GPx, glutathione peroxidase; GSH, glutathione; GSSG, oxidized glutathione; HA, hyaluronic acid; HD, hesperetin derivative; HD-11, hesperidin derivative; HD-16, hesperetin derivative 16; HNE, hydroxynonenal; HSCs, hepatic stellate cells; HYP, hydroxyproline; IFN-γ, interferon-gamma; IGF-1R, insulin-like growth factor (IGF)-1 receptor; IL-1, interleukin-1; IL-10, interleukin-10; IL-18, interleukin-18; IL-1β, interleukin-1β; IL-6, interleukin-6; IL-8, interleukin-8; iNOS, inducible nitric oxide synthase; IRF3, interferon regulatory factor 3; IV-C, collagen type-IV; JNK, c-Jun N-terminal kinase; LATS1, large tumor suppressor kinase 1; LN, laminin; LX-2, human hepatic stellate cell line; MCP-1, monocyte chemoattractant protein-1; MDA, malondialdehyde; miRNAs, microRNAs; MMP-1, matrix metalloproteinase 1; MMP-13, matrix metalloproteinase 13; MMP-2, matrix metalloproteinase 2; MMP-9, matrix metalloproteinase 9; MT1-MMP, membrane type 1-matrix metalloproteinase; NF-κB, nuclear factor kappa B; NK, natural killer; NO, nitric oxide; OPN, osteopontin; PAI-1, plasminogen activator inhibitor-1; p-Akt, phosphorylated protein kinase B; PC, protein carbonyl; PC-III, procollagen type-III; PDGF-BB, platelet-derived growth factor; PDGFRβ, platelet-derived growth factor receptor beta; PI3K, phosphatidylinositol 3-kinase; p-PI3K, phosphorylated phosphatidylinositol-3 kinase; pro-MMP-2, pro-matrix metalloproteinase-2; Ptch1, patched1; PTEN, phosphatase and tension homologue deleted on chromosome ten; Shh, Sonic hedgehog; SIRT3, sirtuin 3; Smad2/3, small mothers against decapentaplegic 2/3; SOD, superoxide dismutase; Sp1, specificity protein 1; SQSTM1, sequestosome 1; STAT3, signal transducer and activator of transcription 3; STING, stimulator of interferon genes; TAA, thioacetamide; TAC, total antioxidant capacity; TBARS, thiobarbituratic acid-reactive substances; TGF-β1, transforming growth factor beta 1; TNF-α, tumor necrosis factor alpha; YAP, Yes-associated protein; α-SMA, alpha smooth muscle actin; γ-GT, γ-glutamyl transpeptidase; TBIL, total bilirubin.

**Table 3:** Pharmacological effects of flavonols in liver fibrosis.

Bioactive Compounds	Cell Lines/ Animal Model	Pharmacological Effects	Reference
Fisetin	Albino Wistar rats	- ↓ serum levels of ALT, AST, ALP, TBIL, and liver index - ↑ the levels of ALB and total protein - ↑ GSH - ↓ MDA - ↓ inflammatory biomarkers: TNF-α, IL-6 - ↓ TGF-β1, Col-1, TIMP-1 - ↑ MMP-9 - suppressed Wnt3a gene expression associated with decreased β-catenin - ↑ GSK-3β levels - ↓ the progress of histologic hepatic fibroplasia - ↓ α-SMA and cyclin D1	[80]
Galangin	LX-2 cells	- inhibited the proliferation of LX-2 cells - induced the apoptosis of LX-2 cells - ↓ the mRNA and protein expression of α-SMA and Col-1 - inhibited PI3K/Akt signaling - ↓ the expression of proteins p-PI3K and p-Akt - triggered the mitochondrial apoptotic pathway by regulating the expression of the Bcl-2 family of proteins - ↑ Bax - ↓ Bcl-2 - ↑ Bax/Bcl-2 ratio - inhibited the Wnt/β-catenin pathway - ↓ the expression of phospho-GSK-3β, total β-catenin, non-phospho(active) β-catenin	[81]
	Sprague-Dawley rats	- ↓ hepatic index - ↓ serum HA and LN levels - ↓ serum ALT and AST levels - ↑ serum level of ALB - ↓ MDA - ↑ SOD and GSH-Px activities - ↓ HYP - alleviated liver damage - ↓ steatosis and hepatic lesions - ↓ α-SMA - ↓ TGF-β	[82]
Isorhamnetin	HSC-T6 cells	- ↓ HSC-T6 activation - ↓ mRNA expression of ColA1 and α-SMA in the activated HSC-T6 cells - suppressed the p-Akt	[83]
	C57 mice	- inhibited liver fibrosis induced by CCl_4_ and BDL in vivo - ↓ ALT and AST - liver HYP levels - ↓ collagen deposition - inhibited massive macrophage recruitment in liver tissues - ↓ the mRNA and protein expression of F4/80 - inhibited HSC activation and ECM deposition - ↓ α-SMA, Col-1 - ↑ PPAR-γ - ↑ MMP-2 - ↓ TIMP-1 - inhibited autophagy in both liver fibrosis mouse models - ↓ Beclin-1 and LC3 - ↓ TGF-β1-activated Smad3 and p38 MAPK signaling pathways - ↓ TGF-β - ↓ p-Smad3 and p-p38 MAPK proteins	[84]
	ICR mice LX-2 cells	- inhibited HSC activation in vitro - ↓ PAI-1, α-SMA, Col1α1 - inhibited TGF-β/Smad signaling pathway - inhibited SBE reporter activity - ↓ Smad3-dependent transcription of SBE reporter genes - blocked TGF-β1-induced phosphorylation of Smad2 and Smad3 - ↑ nuclear Nrf2 levels - ↑ the expression of antioxidant enzymes: GCL and HO-1 - prevented ROS production in LX-2 cells - inhibited liver fibrosis in vivo - ↓ ALT and AST - hepatic degeneration, inflammatory cell infiltration, and collagen accumulation - ↓ the expression of phosphorylated Smad3, TGF-β1, α-SMA, and PAI-1 - ↓ 4-HNE, nitrotyrosine-positive cells - ↑ GSH	[85]
Kaempferol	Sprague-Dawley rats HSC-T6 cells	- ameliorated CCl_4_-induced hepatic fibrosis in rats - ↓ ALT, AST - ↓ α-SMA, Col-1, ASIC1a, VEGF - inhibited the activation and VEGF release of HSCs - ↓ VEGF, α-SMA, Col-1 - inhibited ASIC1a protein expression - promoted the degradation of ASIC1a protein via the ubiquitination pathway - hindered Ca^2+^ influx - inhibited the level of ERS in vitro - ↓ p-eIF2, ATF-4, VEGF - inhibited ERS by targeting ASIC1a in vitro - inhibited HSC activation and ERS by suppressing ASIC1a expression	[86]
	C57BL/6 J mice Primary HSCs	- ameliorated CCl_4_-induced liver fibrosis in vivo - collagen deposition - ↓ α-SMA - ↓ ALT, AST, HYP - suppressed HSC activation in vitro - ↓ α-SMA, Col-1 - promoted HSC activation inhibition via down-regulating Jag1 - ↓ TGF-β, Notch, NF-κB, MAPK/JNK, MAPK/ERK, Wnt - ↓ Hes1, Hes5 - ↓ Jag1 - suppressed HSC activation through miR-26b-5p-mediated Jag1 axis	[87]
	C57BL/6 mice Primary HSCs	- attenuated CCl_4_-induced liver injury and inflammation - ↓ the necroinflammatory scores and collagen deposition in the liver tissue - ↓ ALT, AST - ↓ LN, HA - suppressed HSC collagen synthesis both in vitro and in vivo - ↓ Col1α1 - ↓ collagen deposition - inhibited HSC activation both in vivo and in vitro - ↓ α-SMA - ↓ the phosphorylation of Smad2 and Smad3 - bonded to the ATP-binding site of activin receptor-like kinase 5	[88]
Dihydrokaempferol	C57BL/6 mice HepG2 and LX-2 cells	- attenuated CCl_4_-induced liver injury and hepatic fibrosis in vivo - ↓ ALT, AST - ↓ hepatic index - ↓ α-SMA, Col-1, Col-3 - exhibited antioxidant and anti-inflammatory activity and alleviated hepatocyte DNA damage - ↓ HYP, MDA, H_2_O_2_ - ↑ SOD - ↓ IL-6, IL-1β, TNF-α - suppressed apoptosis of hepatocytes in mice and HepG-2 cells - inhibited the TGF-β1-Smad2/3 and ERK1/2 signaling pathway - ↓ TGF-β1, p-Smad2/3, p-ERK1/2, α-SMA, Col-1, collagen 3 - ↓ the phosphorylation levels of NF-κB P65, ASK1, and JNK - ↓ IL-6, IL-1β, and TNF-α - inhibited PARP-1 activity in hepatocytes by binding to Glu-988 and his-862 residues	[89]
Morin	LX-2 cells Wistar rats	- activated Hippo signaling in cultured LX-2 cells - ↑ Mst1, Lats1 - ↓ Yap, TAZ - suppressed exacerbated TGF-β/Smad signaling in cultured LX-2 cells - ↓ TGF-β1, p-Smad2/3 - ↓ MMP-2, MMP-9, TIMP-1 - ↑ MMP-1 - activated Hippo signaling in vivo - ↑ Mst1, Lats1 - ↓ Yap, TAZ - ↓ TGF-β signaling and attenuated fibrillar collagen deposition in fibrotic rats - ↓ TGF-β1, p-Smad2/3 - ↓ MMP-2, MMP-9, TIMP-1 - ↑ MMP-1 - ↑ HYP - ↓ Col-1, Col-3	[90]
	Sprague-Dawley rats	- ↓ ALT, AST, ALP - ↓ hyperplasia of fiber tissue - ↓ inflammatory cells - ↓ α-SMA, Col-1, Col-3 - ↑ Nrf2, HO-1, NQO1	[91]
	Sprague-Dawley rats Cultured HSCs	- enhanced the expression of PGC-1α - the effect of morin on PGC-1α expression was mediated by AMPK activation - enhanced the activity and the expression of SOD2 via AMP/PGC-1α axis - PGC-1α inhibited the expression of α1 (I) collagen	[92]
	LX-2 cells Wistar rats	- inhibited canonical NF-κB signaling in cultured LX-2 cells - ↓ α-SMA, NF-κB p65 - induced apoptosis in cultured LX-2 cells - ↓ Bcl-2 - ↑ Bax, cyt c - activated caspase-9 and cleaved caspase-3 - induced apoptosis - attenuated liver fibrosis by suppressing NF-κB signaling in vivo - ↓ NF-κBp65 - ↑ IκBα - ↓ α-SMA - ↓ collagen deposition - induced apoptosis in vivo - ↓ Bcl-2 - ↑ Bax, cyt c - activated caspase-9 and cleaved caspase-3	[93]
	Albino rats	- ↓ liver index - ↓ serum levels of ALT, AST, ALP, and TBIL - ↓ oxidative stress - ↓ MDA, NO - ↑ GSH - prevented abnormal collagen deposition - ↓ HYP - attenuated the inflammatory and fibrogenic markers - ↓ TNF-α, iNOS, NF-κB p65	[94]
	LX-2 cells Albino rats	- ↓ the viability of cultured LX-2 cells - induced G1 cell cycle arrest in cultured LX-2 cells - ↓ cyclin D1 - inhibited Wnt signaling in cultured LX-2 cells - ↓ GFAP - ↓ serum levels of AST, ALT, ALP, LDH, and γ-GT in vivo - ↓ MDA, TBARS - ↑ SOD, CAT, GPx, GR - ↓ collagen accumulation - inhibited HSC activation in DEN-induced rats - ↓ GFAP - alleviated DEN-induced liver fibrosis through suppression of Wnt signaling - ↓ GSK-3β, β-catenin, cyclin D1	[95]
	Sprague-Dawley rats	- ↓ fibrosis score - ↓ serum levels of ALT, AST, and bilirubin - ↓ mRNA and protein levels of Col-1, TGF-β1, and α-SMA	[96]
Myricetin	BALB/c mice CFSC-8B cells	- inhibited TGF-β1 or PDGF-BB-induced cell proliferation and migration in CFSC-8B cells - ↓ ECM accumulation induced by TGF-β1 or PDGF-BB in CFSC-8B cells - ↓ α-SMA, Col-1 - suppressed TGF-β1-stimulated phosphorylation of Smad2, AKT, ERK, and P38 in CFSC-8B cells - ↓ PDGF-BB-induced ERK and Akt phosphorylation in CFSC-8B cells - attenuated liver fibrosis induced by CCl_4_ in mice - ↓ degree of liver fibrosis - ↓ ALT, AST - ↓ α-SMA, Col-1 - inhibited the phosphorylation of Smad2, MAPK, Akt, ERK, JNK, and P38	[97]
Myricitrin	BALB/c mice	- ↓ ALT, AST - ameliorated centrilobular necrosis - ↓ hepatic oxidative stress - ↑ GSH, TAC - ↓ 4-HNE, TBARS - ↑ CYP2E1 - suppressed inflammation - ↓ COX-2, TNF-α - inhibited the pro-fibrotic response - ↓ TGF-β1, α-SMA - improved the regeneration of hepatic tissue - ↑ PCNA	[98]
Quercetin	Sprague-Dawley rats	- ameliorated hepatic dysfunction - altered parameters of sphingolipid and pyroptosis pathways - ↓ serum levels of ALT, AST, and ALP - ↑ ALB and total protein levels - ↓ fibrosis and inflammation - ↓ α-SMA, IL-1β, PPAR-γ, TGF-β1, caspase-1, caspase-3 - ↓ SphK1 and NLRP3 - ↓ MDA - ↑ TAC, GSH, Nrf2	[99]
	Wistar rats	- ↓ HDL cholesterol, LDL cholesterol - regulated oxidative stress - ↑ SOD, GSH - ↓ the mediators of the Hh signaling and inflammation: Shh, Ihh, Ptch-1, Smo, Hhip, Gli-3, TNF-α, NF-κβ, and Socs-3 - ↓ hepatic lobule degeneration, the intralobular occurrence of inflammatory cells, and hepatocytic necrosis	[100]
	BALB/c mice Raw 264.7 cells	- attenuated liver inflammation and fibrogenesis in CCl_4_-treated mice - ↓ Col-3 or Col-4 protein expression - ↓ gene expression of Col3α1, Col4α1, CTGF, and TIMP-1 - inhibited HSC activation - inhibited massive macrophage recruitment into the fibrotic livers of CCl_4_-induced mice - ↓ F4/80 and CD68 - inhibited M1 polarization and M1-related inflammatory cytokines in fibrotic livers - ↓ TNF-α, IL-1β, IL-6, MCP-1 - attenuated M2 macrophage polarization and expression of immunosuppressive genes in fibrotic livers - inhibited macrophage activation and M1 polarization in vitro - ↓ the mRNA expression of M1 macrophage markers: TNF-α, IL-1β, IL-6, and NOS2 - inhibited hepatic macrophage activation and suppressed M1 polarization through regulating the expression of Notch1 on macrophages in vivo and in vitro	[101]
	C57 mice	- improved liver fibrosis induced by BDL or CCl_4_ in vivo - ↓ ALT, AST - ↓ HYP - inhibited ECM formation and regulated MMP-9 and TIMP-1 expression - ↓ the levels of serum HA, LN, Col-1, and α-SMA - ↓ Col-1, α-SMA, and TIMP-1 - ↑ MMP-9 - attenuated liver damage by suppressing the TGF-β1/Smads signaling pathway - ↓ TGF-β1 - ↓ p-Smad2 and p-Smad3 - attenuated liver damage via the PI3K/Akt signaling pathway - ↑ PI3K, p-Akt - inhibited autophagy process in BDL- or CCl_4_-induced liver fibrosis - ↓ Beclin-1, LC3 - ↑ P62	[102]
	BALB/c mice RHSteC cells	- ameliorated liver injury, inflammation, and hepatic fibrogenesis induced by CCl_4_ - ↓ ALT, AST - ↓ the mean fibrosis score - ↓ Col-1 - inhibited the activation of HSCs in vivo and in vitro - ↓ α-SMA protein expression - ↓ the expressions of mRNA encoding collagen-α1 (I), TGF-β1, and α-SMA - reduced fibrosis - ↓ HMGB1, TLR2, TLR4 - inhibited the cytoplasmic translocation of HMGB1 in hepatocytes of fibrotic livers - attenuated CCl_4_-induced nuclear translocation of NF-κB p65 and inhibited degradation of IκBα	[103]
	Wistar rats	- ↓ hepatic markers in serum: AST, ALT, GGT - ↓ fibrosis index - ↓ fibrotic area - ↓ collagen - improved gene expression of antioxidant enzymes - ↑ CAT and SOD - inhibited inflammatory markers - ↓ TNF-α, IL-6, NF-κB - ↓ mRNA expression of pro-fibrogenic molecules - ↓ TGF-β1, Col-1, CTGF, TIMP-1 - ↑ anti-fibrogenic molecule gene expression - ↑ MMP-2, MMP-9 - α-SMA - induced HSC apoptosis - ↑ the number of apoptotic cells	[104]

Legend: ↑ increased/up-regulated; ↓ decreased/down-regulated; LDH, lactate dehydrogenase; GFAP, glial fibrillary acidic protein; 4-HNE, 4-hydroxy-2-nonenal; Akt, protein kinase B; ALB, albumin; ALP, alkaline phosphatase; ALT, alanine aminotransferase; AMPK, adenosine monophosphate-activated protein kinase; ASK1, apoptosis signal-regulating kinase-1; AST, aspartate aminotransferase; Bax, Bcl-2-associated X protein; Bcl-2, B-cell lymphoma-2; BDL, bile duct-ligated; CAT, catalase; CCl_4_, carbon tetrachloride; Col-1, collagen 1; Col1α1, collagen type I alpha 1 chain; Col-3, collagen 3; Col3α1, collagen type 3 alpha 1; Col4α1, collagen type 4 alpha 1; COX-2, cyclooxygenase-2; CTGF, connective tissue growth factor; CYP2E1, cytochrome P450 2E1; DEN, diethylnitosamine; ECM, extracellular matrix; ERK, extracellular signal-regulated protein kinase; ERK1/2, extracellular signal-regulated kinases 1/2; GCL, glutamate-cysteineligase; Gli-3, glioma-associated oncogene-3; GPx, glutathione peroxidase; GR, glutathione reductase; GSH, reduced glutathione; GSH-Px, glutathione peroxidase; GSK-3β, glycogen synthase kinase-3 beta; HA, hyaluronic acid; Hes1, hes family bHLH transcription factor 1; Hes5, hes family bHLH transcription factor 5; Hhip, Smo, hedgehog-interacting protein; HMGB1, high-mobility group box 1; HO-1, heme oxygenase-1; HSCs, hepatic stellate cells; HYP, hydroxyproline; Ihh, Indian hedgehog; IL-1β, interleukin-1β; IL-6, interleukin-6; iNOS, inducible nitric oxide synthase; Jag1, Jagged 1; JNK, c-Jun N-terminal kinase; LN, laminin; LX-2, human hepatic stellate cell line; MAPK, mitogen-activated protein kinase; MCP-1, monocyte chemotactic protein-1; MDA, malondialdehyde; miRNAs, microRNAs; MMP-1, matrix metalloproteinase 1; MMP-2, matrix metalloproteinase 2; MMP-9, matrix metalloproteinase 9; NF-κB, nuclear factor kappa B; NO, nitric oxide; NOS2, nitric oxide synthase 2; NQO1, quinone oxidoreductase 1; Nrf2, nuclear factor erythroid 2-related factor 2; PAI-1, plasminogen activator inhibitor-1; p-Akt, phosphorylated protein kinase B; PARP-1, poly ADP-ribose polymerase-1; PCNA, proliferating cell nuclear antigen; PDGF-BB, platelet-derived growth factor; PGC-1α, peroxisome proliferator-activated receptor-γ coactivator-1α; PI3K, phosphatidylinositol-3 kinase; PPAR-γ, peroxisome proliferator-activated receptor-γ; p-Akt, phosphorylated protein kinase B; p-PI3K, phosphorylated phosphatidylinositol-3 kinase; p-Smad3, phosphorylated Smad3; Ptch-1, patched homolog 1; RHSteC, primary rat hepatic stellate cell line; ROS, reactive oxygen species; SBE, Smad binding element; Shh, Sonic hedgehog; Smad2/3, small mothers against decapentaplegic 2/3; Socs-3, suppressor of cytokine signaling 3; SOD, superoxide dismutase; SOD-2, superoxide dimutase-2; SphK1, sphingosine kinase-1; TAC, total antioxidant capacity; TBARS, thiobarbituratic acid-reactive substances; TGF-β1, transforming growth factor beta 1; TIMP-1, tissue inhibitor of metalloproteinases 1; TLR2, toll-like receptor 2; TLR4, toll-like receptor 4; TNF-α, tumor necrosis factor alpha; VEGF, vascular endothelial growth factor; Yap, Yes-associated protein; α-SMA, alpha smooth muscle actin; γ-GT, γ-glutamyl transpeptidase; TBIL, total bilirubin.

**Table 4:** Pharmacological effects of flavones in liver fibrosis.

Bioactive Compounds	Cell Lines/ Animal Model	Pharmacological Effects	Reference
Alpinetin	C57BL/6 mice	- ameliorated the liver injury in mice induced by CCl_4_ administration - ↓ HYP, collagen deposition - ↓ α-SMA, fibronectin, and α1(I) procollagen both at mRNA and protein levels - inhibited the expression of COX-2 and iNOS in vivo - inhibited ROS - ↓ MDA - ↑ GSH, CAT, GSH-Px, SOD - inhibited sinusoidal angiogenesis - ↓ VEGF, VEGFR2, PDGF-βR, HIF-1α - inhibited the activation of the NLRP3 inflammasome - ↓ NLRP3, caspase-1 p20, ASC, mature IL-1β, mature IL-18 - had protective effects against CCl_4_-induced liver fibrosis in mice through the activation of Nrf2 signaling - ↑ protein expression of HO-1, NQO1, GCLM, and GCLC	[108]
Apigenin	Swiss albino mice	- improved hepatic biomarkers - ↓ ALT, AST, TBIL - enhanced oxidative biomarkers - ↑ GSH, MDA, CAT - ↓ IL-1β - , IL-6, TNF-α - ↓ VEGF, CD34	[109]
	LX-2 cells C57 mice	- inhibited proliferation and decreased the viability of LX2 cells - protected the liver against fibrosis induced by CCl_4_ and BDL in mice - ↓ ALT, AST - normalized the structure of portal area - ↓ fibrous tissue hyperplasia and inflammatory cell infiltration - inhibited the activation of HSCs and regulated the balance of TIMP-1 and MMP-2 - ↓ Col-1, α-SMA, IL-1β - ↑ MMP-2, TIMP-1 - alleviated autophagy during liver fibrosis - ↓ Beclin-1 and LC3II/LC3I - ↑ p62 - relieved hepatic fibrosis induced by CCl_4_ and BDL via down-regulating the TGF-β1/Smad3 and p38/PPAR-α pathways - ↓ TGF-β1, p-Smad3, p-p38 - ↑ PPAR-α	[110]
	Wistar rats	- alleviated CCl_4_-induced liver fibrosis - ↓ AST, ALT, ALP, LDH, HYP, TP, TBIL, DB, HA, LN, PCIII, and IV-C - ↓ the mRNA and protein expression of TGF-β1, α-SMA, HIF-1α, FAK/p-FAK, VEGF, iNOS, and p38 MAPK/p-p38 MAPK - ↑ ALB, SOD, and GSH-PX - ↓ MDA	[111]
Baicalin	HSC-T6 cells	- inhibited the proliferation, activation, apoptosis, and cell cycle progression of activated HSC-T6 cells induced by PDGF-BB - ↓ α-SMA - ↑ the number of apoptotic cells - ↑ the number of cells in S phase - ↓ the number of cells in the G0/G1 phase - ↓ EMT of activated HSC-T6 cells induced by PDGF-BB - inhibited the motility and invasive ability of the induced HST-T6 cells - ↑ miR-3595 - miR-3595 regulated the anti-fibrotic effect of baicalin - induced miR-3595 expression that modulated the expression levels of ACSL4 - ↓ ACSL4	[112]
	Sprague-Dawley	- exhibited hepatoprotective effects - ↓ ALT, AST, ALP - suppressed hepatic fibrosis - ↓ HYP - ↓ the area of hepatic fibrosis - suppressed release of inflammatory factors - ↓ TNF-α, TGF-β1 - abrogated the expression of TGF-β1-related proteins and increased the expression of PPARγ mRNA and protein - ↓ the mRNA levels of TGF-β1, Tβ-RI, Tβ-RII, Smad3, α-SMA, and αI(I) collagen - ↑ the expression of PPARγ mRNA and protein	[113]
Baicalein	Sprague-Dawley rats	- ↓ AST, ALT - ↓ HA, LN, PCIII levels - ↓ MMP-2, MMP-9 - ↓ PDGF-β receptor protein levels - ↓ HYP - Col-1, Col-3 - ↓ liver fibrosis	[114]
Chrysin	CD1 mice	- ↓ fibrotic scores - ↓ alleviated the ultrastructure of livers - ↓ Col-1 mRNA expression - restored TIMP-1/MMP balance - ↓ TIMP-1, MMP-2, MMP-3, and MMP-9 mRNA expression - ↑ MMP-1 mRNA expression	[115]
	CD1 mice	- ameliorated hepatic lesions in vivo - inhibited the activation of HSC cells - ↓ α-SMA, TGF-β1 - ↓ Smad 2, Smad 3	[116]
Diosmin	Albino rats	- improved hepatotoxicity biomarkers of fibrotic rats - ↓ AST, ALT γ-GT, TBIL - ↑ ALB, TP serum level - mitigated oxidative stress, enhanced antioxidant defense - ↑ MDA, AOPP, NO levels - ↑ GPx, CAT, GSH - alleviated hepatic inflammatory response - ↓ iNOS, COX-2, NF-κB p65 - abrogated pro-fibrogenic cytokines and stimulated PPAR-γ expression - ↓ α-SMA, TGF-β - ↑ PPAR-γ - ↑ CD36, Arg-1 - represses miR-17-5p/canonical Wnt/β-catenin signaling axis - ↓ miR-17-5p - ↓ Wnt genes (Wnt3a, Wnt10b) - ↓ frizzled receptors (Fzd-1, Fzd-4) - depleted β-arrestin 2, prevented GSK-3β phosphorylation, and destabilized cytoplasmic β-catenin in radiation-induced liver fibrosis - ↓ the protein expression of β-arrestin 2 and p-GSK-3β - ↓ the protein expression of β-catenin, TCF-4, and cyclin-D1	[117]
Eupatilin	LX-2 cells c57BL/6J mice	- represses the activation of HSCs in vitro - ↓ the expression of collagens and α-SMA - inhibited the proliferation of HSCs - ↓ cell viability - ↓ c-Myc, cyclin B1, cyclin D1, CDK6 - inhibited the EMT of HSCs - ↓ N-cadherin - PAI-1 regulated the EMT and activation of HSCs - ↓ PAI-1 - knockdown of PAI-1 using PAI-1-specific shRNA suppressed the levels of Col1α1, α-SMA, and N-cadherin in LX-2 cells - inactivated the - β-catenin signaling pathway to slow down the EMT progression of HSCs in hepatic fibrosis - ↓ the protein level of β - -catenin and its nuclear translocation, while the transcript level of - β-catenin was not affected in LX-2 cells - ameliorated hepatic fibrosis in vivo - ↓ AST - ↓ Col1α1, fibronectin, α-SMA, PAI-1, N-cadherin, - β-catenin	[118]
Isoorientin	Wistar rats	- ↓ ALT, AST, IL-6, TNF-α, MDA, MPO, HA, LN, PCIII, HYP - ↑ SOD, GSH-Px - ↓ α-SMA, TGF-β1	[119]
Isoorientin-2″-O-α-L-arabinopyranosyl	Wistar rats	- improved the liver function - ↓ ALT, AST, ALP, γ-GT - ↓ IL-6, TNF-α, MPO - attenuated oxidative stress - ↓ MDA - ↑ SOD, GSH-Px - inhibited collagen deposition - ↓ serum levels of HA, LN, PCIII, and HYP - induced HSC apoptosis - ↓ Bcl-2 mRNA - ↓ α-SMA, TGF-β1	[120]
Isovitexin	C57BL/6 mice LX2 and JS-1 cells	- ameliorated CCl_4_-induced hepatic fibrosis in vivo - ↓ ALT, AST - ↑ ALB - ↓ hepatic inflammation and alleviated liver injury in vivo - ↓ IL-6, TNF-α - ↑ IL-10 - attenuated oxidative stress in vivo - ↓ ROS level - ↑ SOD, CAT - inhibited HSC activation and ameliorated hepatic fibrosis in vitro - ↓ the cell viability and colony count in LX2 cells - ↓ cell proliferation - ↑ the apoptotic rate of LX2 and JS-1 cells - ↓ collagen deposition in vitro - inhibited the gene and protein expressions of Col-1, Col-3, and α-SMA in LX2 cells - inhibited the PI3K-Akt pathway in vivo and in vitro - ↑ PTEN gene expression - ↓ PI3K, Akt, and mTOR levels - ↑ p-PTEN/PTEN ratio - ↓ p-PI3K/PI3K, p-Akt/Akt, p-mTOR/mTOR - activated autophagy - ↑ the mRNA and protein expression levels of Atg5, Atg7, Beclin-1, and LC3 in vivo - enhanced Atg5 and Atg7 protein expression and the LC3 II/I ratio and inhibited P62 protein levels in LX2 and JS-1 cells - inhibited miR-21 expression by down-regulating m6A modification - ↑ pri-miR-21 level - ↓ the m6A enrichment of pri-miR-21 - attenuated liver fibrosis by modulating the GSH metabolic pathway - ↑ GSH and GSH/GSSG levels - ↓ GSSG levels	[121]
Ligustroflavone	C57BL/6J mice LX-2 cells	- protected against CCl_4_-induced liver injury - ameliorated CCl_4_-induced hepatic dysfunction - ↓ ALT, AST - ↑ ALB - alleviated oxidative injury - ↓ MDA - ↑ GSH-Px, SOD - ameliorated CCl_4_-induced histological damage and liver fibrosis in liver injury - ↓ necrosis of HSCs - ↓ collagen deposition - ↓ HYP - attenuated the expressions of α-SMA and Col-1 in vivo and in vitro - ↓ vimentin and ↑ E-cadherin in vivo - ↓ TGF-β/Smad signaling pathway in vitro - ↓ Tβ-RI, Tβ-RII, p-Smad2, p-Smad3, and Smad4 in LX-2 cells	[122]
Luteolin	HSC-T6 cells Wistar rats	- attenuated liver fibrosis in HSC-T6 cells - ↓ α-SMA and Col1α1 - ↓ the liver fibrosis index level in vivo - ↓ number of inflammatory cells - ↓ collagen deposition - ↓ ALT, AST, ALP - ↑ CCR1, CD59, NAGA - ↓ ITIH3, MKI67, KIF23, DNMT1, P4HA3, CCDC80, APOB, FBLN2	[123]
	HSC-T6 cells Sprague-Dawley rats	- suppressed HSC activation - ↓ α-SMA - ↑ E-cadherin - inhibited proliferation, migration, collagen synthesis, and the expression of fibrosis-related genes in the activated HSCs and HSC-T6 cells stimulated with or without TGFβ1 or PDGF - induced apoptosis of HSC-T6 cells - ↑ caspase 3 activity - ↑ p53 - ↓ Bcl-2, cyclin E, and p-CDK-2 - induced G1 arrest - ameliorated liver fibrosis in vivo - improved the histological changes - ↓ ECM accumulation - ↓ ALT, AST - ↓ α-SMA, Col-1, Col-3, vimentin, and snail - ↑ E-cadherin - induces HSC apoptosis in liver fibrosis induced by BDL - inhibited PDGF- and TGFβ1-simulated phosphorylation of AKT and Smad2/3 pathway in HSCT6 cells - ↓ PDGF-induced phosphorylation of AKT (Ser 473) - ↓ mTOR - ↓ the mTOR substrate p70S6K - ↓ TGF-β1-induced AKT signaling pathway - inhibited TGF-β1-induced Smad2 and Smad3 phosphorylation - suppressed the expression of phosphorylated AKT and Smad2 in vivo - ↓ p-Smad2 and p-AKT	[124]
	Balb/c mice	- ameliorated CCl_4_-induced hepatic fibrosis - ↓ ALT, AST, ALP - ↓ HYP - ↑ GSH - ↓ Cu/Zn SOD activity - ↑ the liver total retinol concentration - ↓ hepatic lesions and hyaline deposits - inhibited HSC activation - ↓ α-SMA, GFAP - ↑ MT I/II - ↑ MMP-9	[125]
Luteoloside	HSC-T6 cells	- regulated the balance of ECM in activated HSC-T6 - ↓ α-SMA, Col-1, TIMP-1/MMP-13 ratio - ↑ protein expression levels of SIRT1 and ERRα - inhibited TLR2/TLR4 pathway - ↓ TLR2 and TLR4 in activated HSC-T6 - ↓ the protein expressions of MyD88, IRAK1, and IRAK4 - alleviated the production of inflammatory cytokines in activated HSC-T6 - ↓ NLRP3, ASC, caspase-1, IL-1β - regulated TLR2/TLR4 and ECM expressions by targeting SIRT1 - ↑ SIRT1 - inhibited the protein expression levels of TLR4, TLR2, NLRP3, caspase-1, IL-1β, and α-SMA - ↓ TLR2, α-SMA - attenuated inflammatory response in HSCs induced by inflammatory factors from activated macrophages - ↓ TLR4, IRAK4, NLRP3, caspase-1, IL-1β, α-SMA	[126]
Nobiletin	C57/BL6J mice	- ameliorated fiber deposition and liver injury in CCl_4_-induced liver fibrosis - ↓ ALT, AST - ↓ α-SMA, fibronectin 1, Col1α1 - ↓ the expression of inflammatory cytokines: TNF-α, IL-6, IL-1β - ↓ TGF-β1 - ↓ NLRP3 and its downstream inflammatory factors IL-18 and IL-1β - alleviated hepatocyte EMT in liver fibrosis - ↓ N-cadherin, vimentin, TIMP-1, TIMP-2 - ↑ MMP-2 - ↓ α-SMA - alleviated inflammatory status and reduced the production of ROS - induced Hippo/YAP pathway to regulate hepatocyte EMT in liver fibrosis - ↓ YAP and inhibited its nuclear translocation - ↓ TEAD2 - ↓ CCN1 and CCN2 - ↑ autophagy and enhanced autophagy flux to ameliorate hepatocyte EMT - ↑ LC3-II expression - ↓ p62 - inhibited EMT in hepatocytes through promoting autophagy flux and thereby facilitating YAP degradation	[127]
Oroxylin A	LX-2 cells ICR mice	- ameliorated CCl_4_-induced liver fibrosis in vivo - ↓ formation of fibrous septa - ↓ reduced collagen deposition - ↓ α-SMA, fibronectin, Col-1 - activated ferritinophagy in HSCs - ferritinophagy was mediated by NCOA4 - ↑ NCOA4 - ↓ FTH1 - ↑ LC3 and Beclin1 - ↓ p62 - promoted iron levels in HSCs and induced lipid ROS accumulation - regulated HSC senescence through ferritinophagy - ↓ p16 and p21 - ↓ cyclin D1, cyclin E1, CDK4, and CDK6 in the presence of siNCOA4 - inhibited telomerase activity in HSCs to a reduced extent in the presence of NCOA4 interference - ↓ α-SMA and Col-1 - the cGAS-STING pathway played an important role in oroxylin A-induced HSC senescence - ↑ SA-β-Gal - inhibited telomerase activity in HSCs to a lesser extent after disrupting the cGAS-STING pathway - promoted the secretion of cytokines like IFN-β by the cGAS-STING pathway to regulate ferritinophagy - ↑ p16 and p21 in LX2 cells - cGAS siRNA - ↓ the expression of NCOA4 - ↓ the expression level of autophagy-related phenotype - ↓ the content of ROS and iron ions in HSCs	[128]
	C57BL/6 mice Primary HSCs	- inhibited inflammation in liver fibrosis in vivo - ↓ NF-κB, α-SMA, IL-1β, IL-6, IL-18, TNF-α, IFN-γ - ↓ the release of inflammatory factors in HSCs - ↓ NF-κB, NLRP3, TNF-α, IL-1β, IL-18 - exerted anti-inflammatory effect by activating autophagy through PI3K/Akt/mTOR signaling - inhibited the phosphorylation of PI3K, AKT, and mTOR - ↑ the conversion of LC3-I to LC3-II - ↑ the protein expression of Atg5-Atg12 and Beclin1/Atg6 - ↓ the expression of P62 - the induction of PI3K/Akt/mTOR signaling impaired the oroxylin-A-mediated anti-inflammatory effect in HSCs - ↓ the expression of TGF-βR and PDGF-βR at mRNA and protein level - ROS accumulation played a pivotal function in the inhibition of PI3K/Akt/mTOR signaling and anti-inflammatory effect of oroxylin A in HSCs - ↓ IL-6, IL-10, IL-18, IL-1β, TNF-α, IFN-γ	[129]
	HSC-T6 cells ICR mice	- protected mouse livers from CCl_4_-induced injury and inflammation accompanied by ERS activation - ↓ ALT, AST, ALP, LDH - ↓ serum levels of IL-6, IL-8, TNF-α - improved morphological changes in liver tissue - ↓ collagen deposition - ↑ HA, LN, PCIII, and CIV in serum - ↓ α-SMA, α1(I) procollagen, fibronectin - inhibited HSC proliferation in vitro and induced cell cycle arrest in S phase - ↓ cyclin A, cyclin E, CDK - ↑ p15, p21, and p27 - ↓ PDGF-β, TGF-β, EGF receptors - induced apoptosis of activated HSCs through caspase activation - ↓ Bcl-2 - ↑ Bax - ↑ cleaved-caspase-9, cleaved-caspase-3, the cleaved form of PARP - ↑ caspase-7 and caspase-8 - inhibited collagen synthesis and induced collagen degradation in activated HSCs - ↓ fibronectin, α-SMA, Col-1 - ↑ MMP-9 - ↓ TIMP-2 - activated the ERS pathway in activated HSCs - ↑ CHOP and calnexin - triggered apoptosis through activation of the ERS pathway - activation of ERS signaling inhibited the accumulation of collagen and alleviated inflammatory reactions in vivo	[130]
	LX2 and LO2 cells ICR mice	- inhibited HSC contraction - blocked aerobic glycolysis in HSCs - ↓ glucose uptake and consumption - ↓ lactate production - ↓ the mRNA and protein expression of HK2, PFK1, and PKM2 - ↓ the intracellular ATP levels - inhibited LDH-A in HSCs - ↓ liver injury and fibrosis, and inhibited HSC glycolysis and contraction in vivo - ↓ the liver/body weight ratio - ↓ ALT, AST, TBIL, IBIL - ↓ HA, LN, PC-III, HYP - ↓ α-SMA, fibronectin, α1(I) procollagen - inhibited sinusoidal capillarization and restored the fenestrae of liver sinusoidal endothelial cells in fibrotic mice - ↓ the mRNA and protein expression of HK2, PFK1, PKM2, and LDH-A - ↓ α-SMA, MLC2 phosphorylation	[131]
	ICR mice Primary mouse HSCs	- alleviated CCl_4_-induced liver injury and fibrogenesis in the mouse model - restored the normal morphology of hepatocytes and blocked the formation of fibrous nodules - ↓ ALT, AST, ALP - ↓ the collagen accumulation - ↓ α-SMA, α1 (I) collagen, fibronectin - inhibited the expression of PDGF-βR and TGFβ-R1 - inhibited PGDF-BB-induced HSC activation in vitro - ↓ α-SMA, desmin, α1(I) collagen, fibronectin - ↓ TGF-β and TNF-α in activated HSCs - promoted autophagy in CCl_4_-induced mouse liver fibrosis - ↑ the expression of autophagy makers: LC3-A/B, Atg3, Atg4, Atg5, Beclin1/Atg6, Atg7, Atg9, Atg12, Atg14 - ↓ the expression of autophagy substrate p62 - promoted autophagy in activated HSCs - ↑ LC3-B, Atg3, Atg5, Beclin1/Atg6, Atg12, Atg14 - ↓ the expression of autophagy substrate p62 - ↑ autophagic vacuoles - inhibition of autophagy by specific inhibitor 3-MA completely abolished oroxylin A-induced anti-fibrosis effect	[132]
Tricin	LI90 cells	- inhibited PDGF-BB-induced cell proliferation by blocking cell cycle progression and cell migration in the human HSC line LI90 and culture-activated HSCs - inhibited PDGF-BB-induced phosphorylation of p38 MAPK - ↓ PDGF-BB-stimulated cell proliferation - inhibited PDGF-BB-induced phosphorylation of PDGF-Rβ, ERK, and Akt in HSCs	[133]
Wogonin	C57BL/6 mice T6, LX-2 cells	- attenuated liver injury in CCl_4_-induced mice - ↓ ALT, AST - ↓ CCl_4_-induced liver fibrosis in vivo - α-SMA and Col1α1 - promoted apoptosis of HSCs in vivo - ↑ cle-caspase3/9 - attenuated liver fibrosis by promoting apoptosis of HSC-T6 cells and LX-2 cells induced by TGF-β1 in vitro - enhanced cle-caspase3 and cle-caspase9 expression and the ratio of Bax/Bcl-2 in T6 cells	[134]

Legend: ↑ increased/up-regulated; ↓ decreased/down-regulated; 3-MA, 3-methyladenine; ACSL4, long-chain-fatty-acid-CoA ligase 4; Akt, protein kinase B; ALB, albumin; ALP, alkaline phosphatase; ALT, alanine aminotransferase; AOPP, advanced oxidative protein product; AST, aspartate aminotransferase; Atg12, autophagy-related gene 12; Atg14, autophagy-related gene 14; Atg3, autophagy-related gene 3; Atg4, autophagy-related gene 4; Atg5, autophagy-related gene 5; Atg6, autophagy-related gene 6; Atg7, autophagy-related gene 7; Atg9, autophagy-related gene 9; Bcl-2, B-cell lymphoma-2; BDL, bile duct-ligated; CAT, catalase; CCl_4_, carbon tetrachloride; CD34, vascular endothelial cell antigen; CDK4, cyclin-dependent kinase 4; CDK6, cyclin-dependent kinase 6; cGAS, cyclic guanosine monophosphate-adenosine monophosphate synthase; CHOP, CCAAT/enhancer-binding protein (C/EBP) homologous protein; CIV, type IV collagen; cle-caspase 3, cleaved caspase-3; cle-caspase 9, cleaved caspase-9; Col-1, collagen 1; Col1α1, collagen type I alpha 1; Col-3, collagen 3; COX-2, cyclooxygenase-2; DB, direct bilirubin; ECM, extracellular matrix; EGF, epidermal growth factor; EMT, epithelial–mesenchymal transition; ERK, extracellular signal-regulated protein kinase; ERRα, estrogen-related receptor alpha; ERS, endoplasmic reticulum stress; FTH1, ferritin heavy chain 1; GCLC, glutamate-cysteine ligase catalytic subunit; GFAP glial fibrillary acidic protein; GPx, glutathione peroxidase; GSH, glutathione; GSH-Px, glutathione peroxidase; GSK-3β, glycogen synthase kinase-3 beta; GSSG, oxidized glutathione; HA, hyaluronic acid; HIF-1α, hypoxia-inducible factor 1-alpha; HK2, hexose kinase 2; HO-1, heme oxygenase-1; HSCs, hepatic stellate cells; HYP, hydroxyproline; IBIL, indirect bilirubin; IFN-β, interferon-beta; IFN-γ, interferon-gamma; IL-18, interleukin-18; IL-1β, interleukin-1β; IL-6, interleukin-6; iNOS, inducible nitric oxide synthase; IV-C, collagen type-IV; LC3-I, microtubule-associated protein 1 light chain 3 beta-I; LC3-II, microtubule-associated protein 1 light chain 3 beta-II; LDH, lactate dehydrogenase; LDH-A, lactate dehydrogenase-A; LN, laminin; MAPK, mitogen-activated protein kinase; MDA, malondialdehyde; MMP-1, matrix metalloproteinase 1; MMP-13, matrix metalloproteinase 13; MMP-2, matrix metalloproteinase 2; MMP-3, matrix metalloproteinase 3; MMP-9, matrix metalloproteinase 9; MPO, myeloperoxidase; MT I/II, metallothionein; mTOR, mammalian target of rapamycin; NCOA4, nuclear receptor coactivator 4; NLRP3, NLR family pyrin domain containing 3; NO, nitric oxide; NQO1, quinone oxidoreductase 1; Nrf2, nuclear factor erythroid 2-related factor 2; PAI-1, plasminogen activator inhibitor-1; p-Akt, phosphorylated protein kinase B; PARP, poly (ADP-ribose) polymerase; p-CDK-2, phosphorylated cyclin-dependent kinase 2; PCIII, procollagen type III; PDGF, platelet-derived growth factor; PDGF-BB, platelet-derived growth factor; PDGF-β, platelet-derived growth factor beta; PDGF-βR, platelet-derived growth factor receptor beta; PFK1, phosphofructokinase 1; p-GSK-3β, phosphorylated glycogen synthase kinase-3 beta; PI3K, phosphatidylinositol 3-kinase; PKM2, pyruvate kinase type M2; p-mTOR, phosphorylated mammalian target of rapamycin; p-p38, phosphorylated p38; PPAR-α, peroxisome proliferator-activated receptor alpha; PPARγ, peroxisome proliferator-activated receptors γ; p-PI3K, phosphorylated phosphatidylinositol 3-kinase; p-PTEN, phosphorylated phosphatase and tension homologue deleted on chromosome ten; p-Smad2, phosphorylated Smad2; p-Smad3, phosphorylated Smad3; PTEN, phosphatase and tension homologue deleted on chromosome ten; ROS, reactive oxygen species; SIRT1, sirtuin 1; SOD, superoxide dismutase; STING, stimulator of interferon genes; TBIL, total bilirubin; TGF-β1, transforming growth factor beta 1; TGF-βR, transforming growth factor β receptor; TIMP-1, tissue inhibitor of metalloproteinases 1; TIMP-2, tissue inhibitor of metalloproteinases 2; TLR2, toll-like receptor 2; TLR4, toll-like receptor 4; TNF-α, tumor necrosis factor alpha; TP, total protein; Tβ-RI, type I receptors for TGF-β; Tβ-RII, type II receptors for TGF-β; VEGF, vascular endothelial growth factor; VEGFR, vascular endothelial growth factor receptor; VEGFR2, vascular endothelial growth factor receptor 2; YAP, Yes-associated protein; α-SMA, alpha smooth muscle actin; γ-GT, γ-glutamyl transpeptidase.

**Table 5:** Pharmacological effects exerted by flavonones in liver fibrosis.

Bioactive Compounds	Cell Lines/ Animal Model	Pharmacological Effects	Reference
Ampelopsin	ICR mice Primary mouse HSC cells	- attenuated CCl_4_-induced liver dysfunction - ↓ ALT, AST - suppressed CCl_4_-induced hepatic fibrosis - ↓ collagen deposition, ECM deposition, and α-SMA - inhibited the HSC activation in vitro - ↓ α-SMA, MMP-9 - ↑ TIMP-1 - regulated SIRT1/TGF-β1/Smad3 signaling pathway in liver fibrosis - ↑ SIRT1 - ↓ TGF-β1, p-Smad3 - regulated SIRT1/TGF-β1/Smad3 signaling pathway in cultured HSCs - ↑ SIRT1 - ↓ TGF-β1, p-Smad3 - ↓ TGF-β1, p-Smad3, Col-1, and α-SMA in cultured HSCs - promoted CCl_4_-induced autophagy in the livers of mice - ↑ LC3-II and Beclin-1 in cultured HSCs - ↓ α-SMA and Col-1 in cultured HSCs - PI3K/AKT/mTOR pathway was involved in the anti-fibrotic effect of ampelopsin - ↓ the phosphorylation of AKT and mTOR in cultured HSCs	[135]
Naringin	Balb/c mice Primary mouse HSCs	- suppressed activation of primary mouse HSCs - reversed activated HSCs to quiescent cells - ↓ Col-1 and α-SMA - the suppressive effect of naringin on HSC activation was enhanced by inhibition of autophagy - ↑ Beclin1, LC3-II - ↓ p62 - ↓ Col-1 and α-SMA - suppressed activation of HSCs through mTOR pathway - ↓ phosphor-mTOR (Ser2481, Ser2448), phosphor-p70S6K (Thr389), and phosphor-S6 (Ser235/236)	[136]
Pinocembrin	Wistar rats	- restored liver transaminases and total cholesterol to normal levels - ameliorated oxidative stress injury - ↑ GSH, SOD - ↓ MDA - ↑ Nrf2 and HO-1 - alleviated pro-inflammatory cytokines - ↓ TNF-α, NF-κB - ↓ the markers of fibrosis - ↓ Col-1, α-SMA, TGF-β, p-Smad 2/3	[137]
	Human immortalized HSC LX-2 cells Rat immortalized HSCs HSC-T6 cells	- inhibited HSC activation - ↓ Col-1 and α-SMA in both LX-2 and HSC-T6 cells - suppressed HSC activation through inhibiting TGF-β-Smad signaling pathway - ↓ TGF-β1 production and secretion - suppressed Smad phosphorylation and activation - PI3K-Akt signaling was involved in the anti-fibrotic effect of PIN - ↓ the phosphorylation levels of PI3K and Akt in both LX-2 and HSC-T6 cells - ↑ SIRT3 expression, which deacetylated and activated SOD-2 to enhance ROS clearance, resulting in inactivation of HSCs - activated GSK-3β to promote Smad degradation in HSCs - ↓ p-GSK-3β - suppressed HSC activation through SIRT3-TGF-β-Smad signaling pathway	[138]

Legend: ↑ increased/up-regulated; ↓ decreased/down-regulated; Akt, protein kinase B; ALT, alanine aminotransferase; AST, aspartate aminotransferase; CCl_4_, carbon tetrachloride; Col-1, collagen 1; ECM, extracellular matrix; GSH, glutathione; GSK-3β, glycogen synthase kinase-3 beta; HO-1, heme oxygenase-1; HSCs, hepatic stellate cells; LC3-II, autophagy-related protein microtubule-associated protein light chain three II; MDA, malondialdehyde; MMP-9, matrix metalloproteinase 9; mTOR, mammalian target of rapamycin; NF-κB, nuclear factor kappa B; Nrf2, nuclear factor erythroid 2-related factor 2; p-GSK-3β, phosphorylated glycogen synthase kinase-3 beta; PI3K, phosphatidylinositol-3 kinase; p-Smad 2, phosphorylated Smad 2; p-Smad 2/3, phosphorylated Smad 2/3; p-Smad3, phosphorylated Smad3; ROS, reactive oxygen species; SIRT1, silent mating-type information regulation 2 homolog 1; SIRT3, silent mating-type information regulation 2 homolog 3; SOD, superoxide dismutase; SOD-2, superoxide dimutase-2; TGF-β1, transforming growth factor beta 1; TIMP-1, tissue inhibitor of metalloproteinases 1; TNF-α, tumor necrosis factor alpha; α-SMA, alpha smooth muscle actin.

**Table 6:** Pharmacological effects of isoflavones in liver fibrosis.

Bioactive Compounds	Cell Lines/ Animal Model	Pharmacological Effects	Reference
Calycosin	C57BL/6 mice	- suppressed the liver index in CCl_4_-induced hepatic fibrosis mice - inhibited oxidative stress - ↓ AST, ALT, MDA - ↑ SOD - ↓ the scores of collagen deposition and liver fibrosis - inhibited collagen synthesis - ↓ α-SMA, Col-1, HYP - regulated the expressions of MMP-1 and TIMP-1 in CCl_4_-induced hepatic fibrosis mice - ↑ MMP-1 - ↓ TIMP-1 - ↑ the MMP-1/TIMP-1 ratio - regulated the expression of estrogen receptors in CCl_4_-induced hepatic fibrosis mice - ↑ Erβ - activated JAK2-STAT3 pathway - ↑ p-JAK2/JAK2, p-STAT3/STAT3	[139]
Genistein	LX2 cells Wistar rats	- inhibited cell viability and proliferation and induced cell cycle arrest at G0/G1 phase in LX2 cells - ameliorated liver injury and the collagen deposition in rats with DMN-induced fibrosis model - ↓ AST, ALT, ALP, TBA - ↓ HYP - suppressed the expression levels of HSC activation marker α-SMA and Col1A1 in vivo and in vitro - ↓ the mRNA expression levels of MMP-2/9 and TIMP-1 in vivo - regulated inflammatory infiltration and macrophage functional properties in vivo - ↓ the mRNA expression levels of IL-1β, IL-6, TNF-α, and MCP-1 - ↓ CD68 - ↑ CD163, CD206 - inhibited the JAK2/STAT3/Socs-3 signaling pathway - ↓ p-JAK2/JAK2, p-STAT3/STAT3, and Socs-3 protein in vivo and in vitro	[140]
	Wistar rats	- ameliorates D-GalN-induced functional and histological damage - inhibited the activation of HSCs - ↓ α-SMA - ↓ the accumulation of collagen matrix - ↓ TGF-β, Col-1, Col-3, HYP - ↓ nitrotyrosine - inhibited TGF-β/Smad signaling - ↓ TGF-β, Smad 2/3	[141]
	SPF-Wistar rats	- ↓ the plasma alcohol concentration - attenuated the activity of hepatic enzymes ADH and ALDH - ↓ AST, ALT - ↓ the levels of inflammatory mediators: IL-6, TNF-α, MPO, NF-κB - ↑ SOD, GSH-Px, GSH-Rd, CAT - ↓ MDA - inhibited collagen deposition and ↓ pathological tissue damage: HA, LN, and PCIII - attenuated the degree of liver fibrogenesis and the formation of pseudo-lobulus - ↓ inflammatory cell infiltration	[142]
	Wistar rats	- ↓ hepatic collagen - improved liver function - ↓ the amount of collagen fibers and the extent of necrotic areas - ↑ the capacity of the liver to degrade Col-1 and Matrigel - ↑ the number of uPA-immunoreactive cells - ↓ the number of fiber septa in pericentral and perisinusoidal areas - ↓ activation of HSCs - ↓ the number of collagen fibers - ↓ AST, ALT, TBIL	[143]
Glabridin	JS1 cells C57BL/6 mice	- ameliorated the liver injury in CCl_4_-treated mouse livers - ↓ HA, ALT, AST - ↓ the ratio of liver/body weight of mice - inhibited the liver fibrosis in vivo - ↓ collagen deposition - ↓ HYP, mRNA, and protein expression of α-SMA, fibronectin, and α1(I)procollagen - ↑ mRNA and protein expression of PPARγ in vivo - inhibited the inflammation and oxidative stress in vivo - ↓ IL-6, IL-1β, TNF-α - ↑ IL-10 - ↓ iNOS - ↓ MDA - ↑ GSH, T-AOC - ↓ the cell viability of PDGF-BB-stimulated JS-1 cells - inhibited the protein expression of α-SMA, fibronectin, and α1(I)procollagen - ↑ the expression of PPARγ in stimulated JS-1 cells - disruption of PPARγ attenuated the anti-inflammatory and antioxidative stress effects of glabridin in stimulated JS-1 cells	[144]
Puerarin	Sprague-Dawley rats	- alleviated inflammation and fibrosis in TAA-induced liver fibrosis in rats - ↓ histopathological changes and collagen fibers - ↓ HYP - ↓ the protein expression levels of Col-1 and fibronectin - alleviated TGF-β1 expression and HSC activation - ↓ TGF-β1, α-SMA - inhibited the ERK1/2 signaling pathway - ↓ p-ERK1/2 - ↓ the ratio of p-ERK1/2 to ERK1/2	[145]
	Wistar rats	- ↓ HA level in blood and the HYP level in liver - ↓ the areas of liver fibrosis in rats - ↓ the mRNA levels of Col-1, Col-3, Wnt, and β-catenin - ↓ the protein levels of Wnt1 and β-catenin	[146]
	C57BL/6J mice	- protected against CCl_4_-induced chronic liver injury - ↓ the serum ALT, AST, ALB, and TBIL levels - attenuates CCl_4_-induced chronic liver fibrosis - ↓ α-SMA, Col-1, TGF-β, CTGF - inhibited the NF-κB signaling pathway - inhibited ROS production and mitochondrial dysfunction in vivo - ↓ 4-HNE - ↑ mtDNA number - ↑ complex I and II activities - ↓ protein expression of cytosolic cytochrome C - ↓ caspase 3 - protected against CCl_4_-induced liver lesion via modulation on PARP-1 - ↓ PARP-1	[147]
	Wistar rats	- ameliorated the liver metabolic function - ↓ ALT, AST, TBIL - ↑ ALB, TP - ↓ ECM deposition - ↓ Col-3, LN, HA - alleviated the degree of fibrogenesis and ameliorated hepatocellular injury - attenuated the accumulation of collagen - ↓ HYP, PCIII, Col-1 - regulated the expression of TIMP-1 and MMP-2 at the mRNA level - ↑ MMP-2 - ↓ TIMP-1 - ↑ PPAR-γ - ↓ p-PI3K, p-Akt	[148]
	Wistar rats	- ↓ ALT, AST, ALB, TP - restored the hepatic morphology - ↓ TNF-α and NF-κB expressions at protein level - ↑ SOD - ↓ MDA - ↓ TGF-βl - the mRNA level of iNOS	[149]
	Sprague-Dawley rats HSC-T6 cells	- enhanced the survival rate of DMN-treated rats - ↓ the activity of functional liver enzymes - ↓ ALT, AST - reversed the effect of increased collagenic accumulation - ↓ the serum levels of HA, LN, PCIII, and CIV - ↓ visceral indices - ↓ liver index and thymus/spleen indices - alleviated liver fibrosis - ↓ the area of the collagen fibers and the infiltration of inflammatory cells - restored the hepatic parenchyma - ↑ number of hepatocytes - ↓ collagen deposition - ↓ HYP, Col-1 - ↓ protein expression levels of TGF-β1, Smad2, Smad3, α-SMA, and TIMP-1 - ↑ protein expression levels of Smad7 and MMP-1 - inhibited the proliferation of HSC-T6 cells - ↓ the protein levels of TβRI, Smad2, and Smad3 - ↑ the protein levels of Smad7	[150]
	Wistar rats	- ↓ serum ALT, AST - ↑ apoptosis of activated HSCs - ↓ Bcl-2 mRNA	[151]
Soy isoflavone	Sprague-Dawley rats	- ↓ the amount of collagen fibers and the extent of necrotic areas - ↓ collagen fibers around the HSCs following high-dose soy isoflavone administration - ↓ PDGF-BB, TIMP-1 - ↓ α-SMA and TGF-β1	[152]
Tectorigenin	Sprague-Dawley rats	- improved the histological scores - ↓ the average severity scores for liver fibrosis - ↓ ALT, AST - ↑ ALB, the ratio of albumin to globulin (A/G) - ↓ HA, LN, and PIIIP - ↓ HYP - ↓ MDA - ↑ SOD and GSH-Px	[153]

Legend: ↑ increased/up-regulated; ↓ decreased/down-regulated; 4-HNE, 4-hydroxy-2-nonenal; A/G, the ratio of albumin to globulin; ADH, alcohol dehydrogenase; ALB, albumin; ALDH, aldehyde dehydrogenase; ALP, alkaline phosphatase; ALT, alanine aminotransferase; AST, aspartate aminotransferase; Bcl-2, B-cell lymphoma-2; CAT, catalase; CCl_4_, carbon tetrachloride; CIV, type IV collagen; Col-1, collagen 1; Col1A1, collagen type I alpha 1; Col-3, collagen 3; CTGF, connective tissue growth factor; D-GalN, D-Galactosamine; DMN, dimethylnitrosamine; ERK1/2, extracellular signal-regulated kinases 1/2; ERα, estrogen receptor α; ERβ, estrogen receptor β; GSH-Px, glutathione peroxidase; GSH-Rd, glutathione reductase; HA, hyaluronic acid; HSCs, hepatic stellate cells; HYP, hydroxyproline; IL-1β, interleukin-1β; IL-6, interleukin-6; iNOS, inducible nitric oxide synthase; JAK2, Janus kinase 2; LN, laminin; MCP-1, monocyte chemotactic protein-1; MDA, malondialdehyde; MMP-1, matrix metalloproteinase 1; MMP-2, matrix metalloproteinase 2; MMP-9, matrix metalloproteinase 9; MPO, myeloperoxidase; mtDNA, mitochondrial DNA; NF-κB, nuclear factor kappa B; p-Akt, phosphorylated protein kinase B; PARP-1, poly (ADP-ribose) polymerase 1; PCIII, type III precollagen; PDGF-BB, platelet-derived growth factor; p-ERK1/2, phosphorylated extracellular signal-regulated kinases 1/2; PI3K, phosphorylated phosphoinositide 3-kinase; PIIIP, procollagen III N-terminal peptide; p-JAK2, phosphorylated Janus kinase 2; PPARγ, peroxisome proliferator-activated receptors γ; p-STAT3, phosphorylated signal transducer and activator of transcription 3; ROS, reactive oxygen species; Socs-3, suppressor of cytokine signaling 3; SOD, superoxide dismutase; STAT3, signal transducer and activator of transcription 3; TAA, thioacetamide; T-AOC, total antioxidant capacity; TBA, total bile acid; TBIL, total bilirubin TGF-β, transforming growth factor beta; TGF-β1, transforming growth factor beta 1; TIMP-1, tissue inhibitor of metalloproteinase-1; TNF-α, tumor necrosis factor alpha; TP, total protein; TβRI, TGF-β receptor type I; uPA, urokinase-type plasminogen activator; α-SMA, alpha smooth muscle actin.

**Table 7:** Pharmacological effects of anthocyanidins in liver fibrosis.

Bioactive Compounds	Cell Lines/ Animal Model	Pharmacological Effects	Reference
Anthocyanins	C57BL/6J mice Mice HSC cell line	- attenuated liver fibrosis progression in mice - ↓ ALT and AST - inhibited the hepatic inflammatory response and macrophage polarization - inhibited the activation and migration of HSCs - ↓ Col-1 and α-SMA - reversed the blocked autophagic flux induced by PDGF or CCl_4_ - ↑ TFEB	[157]
Anthocyanins from *Aronia Melanocarpa* Elliot	HSC-T6 cells	- inhibited the proliferation cells and reduced the activation of HSC-T6 cells - inhibit the expressions of AST, ALT, ALP, and TBIL - improved the expressions of TP and ALB - inhibited the secretion of inflammatory cytokines IL-1, IL-6, TNF-α, and COX-2 - inhibited the expression of TGF-β1, p-Smad2, α-SMA, and Col-1	[158]
Anthocyanins from blueberry	C57BL/6J mice	- prevented CCl_4_-induced liver damage - ↓ ALT, AST - protected against hepatic oxidative stress induced by CCl_4_ - ↓ MDA - attenuated CCl_4_-induced hepatic inflammation - ↓ CXCL2, MIP-2, MCP-1, and IL-1β - modulated macrophage subsets in CCl_4_-induced liver injury - attenuated CCl_4_-induced hepatic fibrosis - ↓ α-SMA - ↑ MMP-9 - ↓ PCNA, TIMP-1 - attenuated HSC activation - ↓ α-SMA, Col-1, TGFβ-1, and CTGF	[159]
	HSC-T6 cells SD rats	- inhibited the proliferation of HSC-T6 cells - ↑ the apoptosis of HSC-T6 - ↓ the protein expression of α-SMA Col-1 and TIMP-1 in vitro - ↑ acH3K9, acH3K14, and acH3K18 - ↓ ALT, AST - ↓ HA, CIV - ↓ the grade of liver fibrosis	[154]
Cyanidin-3-O-β-glucoside	C57BL mice	- exhibited a protective effect on liver injury and fibrosis - suppressed the activation of HSCs - ↓ α-SMA, desmin, MMP-2, and MMP-9 - regulated oxidative stress and apoptosis in liver - ↓ MDA - ↑ GSH, SOD - ameliorated apoptosis of hepatic cells - ↓ ALT, AST - suppressed leukocyte recruitment but had no significant effect on the activation of Kupffer cells - ameliorated the infiltration of inflammatory cells such as neutrophils and leukocytes - suppressed the production of pro-inflammatory cytokines - ↓ TNF-α, IL-17 - ↑ the level of anti-inflammation cytokine IL-10 - ↓ liver growth factors and MCP-1 - ↓ TGF-β1 and PDGF	[160]
Delphinidin	Balb/C mice	- ↓ ALT, AST - attenuated oxidative stress - ↓ HYP - ↓ hepatic lesions, hepatocyte ballooning, and fatty degeneration - ↓ collagen deposits - ↓ α-SMA, TNF-α, TGF-β1 - ↑ MT I/II - ↑ MMP-9	[161]
Malvidin	HSC-T6 cells	- inhibited HSC-T6 cell proliferation - destroyed HSC-T6 cell morphology - induced HSC-T6 cell apoptosis - triggered HSC-T6 cell ROS generation - ↑ MDA - ↓ SOD, GSH/GSSG ratio - induced apoptosis through an ERS pathway and a mitochondrial pathway - ↑ caspase-12, GRP78, CHOP - ↓ Bcl-2 - ↑ Bax, caspase-3	[162]
Pelargonidin	C57BL/6J mice LX-2 cells	- mitigated the liver weights and relative liver weights compared to the CCl_4_-treated group in mice - ameliorated the hepatic injury markers in vivo - ↓ ALT, AST, ALP - attenuated liver damage and collagen deposition in vivo - suppressed α-SMA, Col-1, and TIMP-1 expression in vivo - inhibited NLRP3 activation and inflammation in CCl_4_-induced mice - ↓ TNF-α, IL-1β, IL-6 - attenuated oxidative stress through Nrf2 activation in vivo - ↓ MDA - ↑ GSH, SOD - ↑ Nrf2 in the nucleus - suppressed the expression of fibrosis markers in TGF-β-activated LX-2 cells - ↓ α-SMA, COL1A1, TIMP-1, Serpin E1/PAL-1 - attenuated cellular oxidative stress and inflammation through Nrf2 activation in TGF-β-activated LX-2 cells - ↑ the Nrf2 protein level in the nucleus - ↓ the mRNA levels of NLRP3, caspase 1, and IL-1β	[163]

Legend: ↑ increased/up-regulated; ↓ decreased/down-regulated; ALB, albumin; ALP, alkaline phosphatase; ALT, alanine aminotransferase; AST, aspartate aminotransferase; Bax, Bcl-2-associated X protein; Bcl-2, B-cell lymphoma-2; CCl_4_, carbon tetrachloride; CHOP, CCAAT/enhancer-binding protein (C/EBP) homologous protein; CIV, type IV collagen; Col-1, collagen 1; Col1A1, collagen type I alpha 1; COX-2, cyclooxygenase-2; CTGF, connective tissue growth factor; CXCL2, chemokine (C-X-C motif) ligand 2; ERS, endoplasmic reticulum stress; GRP78, glucose-regulated protein 78; GSH, glutathione; GSSG, oxidized glutathione; HA, hyaluronic acid; HSCs, hepatic stellate cells; HYP, hydroxyproline; IL-1, interleukin-1; IL-10, interleukin-10; IL-17, interleukin-17; IL-1β, interleukin-1 β; IL-6, interleukin-6; MCP-1, monocyte chemoattractant protein-1; MDA, malondialdehyde; MIP-2, macrophage inflammatory protein 2; MMP-2, matrix metalloproteinase 2; MMP-9, matrix metalloproteinase 9; MT I/II, metallothionein; NLRP3, NLR family pyrin domain containing 3; Nrf2, nuclear factor erythroid 2-related factor 2; PCNA, proliferating cell nuclear antigen; PDGF, platelet-derived growth factor; p-Smad2, phosphorylated Smad2; SOD, superoxide dismutase; TBIL, total bilirubin; TFEB, transcription factor EB; TGF-β1, transforming growth factor beta 1; TIMP-1, tissue inhibitor of metalloproteinases 1; TNF-α, tumor necrosis factor alpha; TP, total protein; α-SMA, alpha smooth muscle actin.

**Table 8:** Pharmacological effects of chalcones in liver fibrosis.

Class of Chalcones	Bioactive Compounds	Cell Lines/ Animal Model	Pharmacological Effects	Reference
Chalcones	Butein	Sprague-Dawley rats	- ↓ ALT, AST - ↓ HYP - ↓ MDA - ↓ alpha1(I) collagen and TIMP-1 mRNA	[164]
	Isobavachalcone	SPF-grade SD rats HSC-T6 cells	- attenuated CCl_4_-induced liver fibrosis - ↓ HYP - ↓ ALT, AST - inhibited CCl_4_-induced HSC activation - ↓ α-SMA and Col-1 - attenuated CCl_4_-induced inflammatory response and oxidative stress - ↓ TNF-α, IL-1β, IL-6 - ↑ SOD and GSH - ↓ MDA - activated Nrf2/HO-1 pathway in vivo - ↓ HO-1, NQO-1 - ↓ NF-κB, TNF-α, IL-1β, IL-6 - inhibited TGF-β1-induced activation of HSC-T6 cells - ↓ α-SMA, Col-1 - attenuated inflammatory response and oxidative stress in vitro - ↓ TNF-α, IL-1β, IL-6 - ↑ SOD, GSH - ↓ MDA - the anti-fibrogenic effects were mediated through the Nrf2/HO-1 pathway - ↓ NF-κB - ↓ α-SMA, Col-1 - the Nrf2 inhibitor (ML385) attenuated the effect of IBC on inhibiting the activation of quiescent HSCs	[165]
	Trans-chalcone	Wistar rats	- ↓ ALT, AST, ALP, IBIL, TBIL - ↓ tissue collagen level - ↓ TGF-β1 - ↓ TBARS, hepatic nitrite/nitrate - ↑ GSH - ↓ TNF-α - ↓ inflammatory cell infiltration - reverted hepatic necrosis - induced regenerative changes in hepatocytes	[166]
Prenylated chalcones	Xanthohumol	BALB/c mice	- ↓ ALT, AST - inhibited the pro-inflammatory and pro-fibrogenic hepatic gene expression - ↓ TNF-α, IL-1α, MCP-1, ICAM-1, NF-κB - ↓ TGF-β, Col-1, TIMP-1 - inhibited the activation of HSCs - ↓ α-SMA	[167]
		Primary human hepatocytes (PHHs) and HSCS BALB/c mice	- inhibited the activation of HSCs in vitro - ↓ α-SMA, Col-1 - induced apoptosis in activated HSCs in vitro - inhibited NF-κB activity and pro-inflammatory gene expression of activated HSCs in vitro - ↓ NF-κB, MCP-1 - inhibited pro-inflammatory gene expression of hepatocytes - ↓ IL-8 - ↓ hepatic inflammation and expression of pro-fibrogenic genes in a murine model of NASH	[168]
Dihydrochalcones	Phloretin	LX-2 cells C57BJ6 mice	- ↓ the fibrogenic marker expression - ↓ α-SMA, Col-1, TGFβ-1 - inhibited cell proliferation, increased apoptosis expression induced by succinate in HSCs - inhibited succinate-induced migration and contraction of HSCs - ↓ TIMP-1, p-MLC2 - ↓ succinate-induced aerobic glycolysis in activated HSCs - ↓ GLUT-1, LDHA, HK II - improved liver fibrosis induced by a sodium succinate diet in vivo - ↓ ALT, triglycerides - ↓ α-SMA, Col-1 - ↓ expression of glycolytic markers in the livers of mice with sodium succinate diet-induced liver fibrosis - ↓ GLUT-1, LDHA	[169]
	Icariin	Mouse primary HSCs C57BL/6 J mice	- inhibited EMT, fibrogenesis, cell growth, and migration of HSCs, and promoted the apoptosis of HSCs - ↑ E-cadherin, GFAP - ↓ desmin, vimentin, α-SMA, Col1A1 - ↓ the cell growth - enhanced the caspase-3 activity of HSCs - exerted hepatoprotective effects on CCl_4_ model in mice - ↓ collagen, α-SMA - ↑ E-cadherin, GFAP - ↓ desmin, vimentin - exerted protective effects on liver fibrosis through miR-875-5p - ↑ miR-875-5p	[170]
		Wistar rats	- ↓ ALT, AST - ↑ ALB - ↓ the number of inflammatory cells - ↓ hepatic collagen deposition - ↓ Col-1α mRNA, HYP - presented anti-angiogenic effect - ↓ mRNA of Ang-1 - ↓ protein expression of VEGF, PDGF-β, and CTGF - ↓ HMGB1, TGF-β, Beclin-1 mRNA - ↑ BAMBI mRNA - exhibited anti-autophagic activity - ↑ mTOR and p70S6 kinase expression - ↓ TLR4, NF-κB, IL-1β, COX-2	[171]
	Icaritin	Wistar rats HSC-T6 and LX-2	- inhibited the growth of activated HSCs - induced the apoptosis of activated HSCs - ↑ Bak-1, Bmf, Bax - ↓ Bcl-2 expression - ↓ AST, ALT, TP, and A/G in vivo - ameliorated the development of liver fibrosis in rats - ↓ HYP, Col-1 - ↓ number of activated HSCs in vivo - ↓ α-SMA	[172]

Legend: ↑ increased/up-regulated; ↓ decreased/down-regulated; A/G, the ratio of albumin to globulin; ALB, albumin; ALP, alkaline phosphatase; ALT, alanine aminotransferase; Ang-1, angiopoietin-1; AST, aspartate aminotransferase; BAMBI, “bone morphogenetic protein” activin membrane-bound inhibitor; Bax, Bcl-2-associated X protein; CCl_4_, carbon tetrachloride; Col-1, collagen 1; Col1A1, collagen type I alpha 1; Col-1α, collagen-1 alpha; COX-2, cyclooxygenase-2; CTGF, connective tissue growth factor; EMT, epithelial–mesenchymal transition; GFAP, glial fibrillary acidic protein; GLUT-1, glucose transporter type 1; GSH, glutathione; HK II, mitochondrial-bound hexokinase (HK) II; HMGB1, high-mobility group box 1; HO-1, heme oxygenase-1; HSCs, hepatic stellate cells; HYP, hydroxyproline; IBC, isobavachalcone; IBIL, indirect bilirubin; ICAM-1, intercellular adhesion molecule-1; IL-1α, interleukin-1α; IL-1β, interleukin-1β; IL-6, interleukin-6; IL-8, interleukin-8; LDHA, lactate dehydrogenase A; MCP-1, monocyte chemoattractant protein-1; MDA, malondialdehyde; mTOR, mammalian target of rapamycin; NASH, non-alcoholic steatohepatitis; NF-κB, nuclear factor kappa B; NQO-1, NAD(P)H quinone dehydrogenase 1; Nrf2, nuclear factor erythroid 2-related factor 2; PDGF-β, platelet-derived growth factor beta; SOD, superoxide dismutase; TBARS, thiobarbituratic acid-reactive substances; TBIL, total bilirubin; TGF-β1, transforming growth factor beta 1; TIMP-1, tissue inhibitor of metalloproteinases 1; TLR4, toll-like receptors 4; TNF-α, tumor necrosis factor alpha; TP, total protein; VEGF, vascular endothelial growth factor; α-SMA, alpha smooth muscle actin; p-MLC2, phosphorylated myosin light chain 2.

**Table 9:** The main pharmacological effects exerted by polyphenol-based drug delivery systems in liver fibrosis.

Polyphenolic Compounds	Active Ingredient	Formulations	Experimental Model	Pharmacological Effects	Reference
Flavonols	Galangin	Galangin delivered by retinoic acid-modified nanoparticles	C57BL/6 mice	- ↓ ALT, AST - ↓ HYP - ↓ HA, LN, PCIII, Col IV - ↓ fibrosis index	[216]
	Quercetin	Theranostic quercetin nanoparticle	BABL-c mice HSC cells	- inhibited proliferation of activated HSCs - attenuated the fibrotic level of the fibrotic tissue, the appearance of degenerated hepatocytes, and inflammatory cell infiltration - ↓ HYP - ↓ ALT, AST - ↓ the protein expression of α-SMA	[217]
Flavones	Chrysin	CHR-HPBCD, CHR-RAMEB nanocomplexes	CD1 mice	- alleviated CCl_4_-induced liver fibrosis collagen deposition, and ultrastructural changes - ↓ Col-1 - ↓ the score of liver fibrosis - inhibited the activation of HSCs - ↓ α-SMA - ↓ the TGF-β1/Smad signaling pathway - ↓ TGF-β1, Smad2, Smad3 - ↑ Smad7 - ↓ the NF-κB-mediated inflammatory pathway - ↓ NF-κB, TNF-α, IL-6 - modulated ECM by TIMP-1/MMP balance - ↓ MMP-2, MMP-3, MMP-9, TIMP-1 - ↑ MMP-1 - modulated pro-fibrotic and anti-fibrotic miRNA expression	[218]
		CHR-HPBCD, CHR-RAMEB complexes	Huh7 and LX2 cells	- the 1:1 CHR-RAMEB pretreatment avoided p65 translocation - the 1:2 CHR-RAMEB complex - ↑ ORAC values - improved SOD activity - produced the highest stimulation of GPx activity - ↓ α-SMA expression at lower concentration than CHR-HPBCD	[219]
	Luteolin	Luteolin-loaded exosomes derived from bone marrow MSCs	Sprague-Dawley rats	- exhibited anti-fibrotic activity - ↓ the % relative liver weight - ↓ ALT, AST, ALP - ↓ TNF-α - ↓ HYP, TGF-β, MMP-2 - restored the liver architecture - ↓ collagen deposition	[220]
Flavanones	Hesperidin	Hesperidin-loaded liposomes	Wistar rats	- ↓ ALT, AST, and ALP - ↑ ALB - improved the liver histological architecture, with restoration of normal hepatocytes and central veins - ↓ TGF-β1	[221]
	Naringenin	Naringenin-loaded multifunctional nanoparticles	Wistar albino rats	- ↓ CCl_4_-induced liver fibrosis - reversed liver damage - ↓ ALP, AST, TBIL - ↓ the pro-inflammatory cytokines TNF-α, IL-1b, and IL-6 - improved pro-MMP-2 and -MMP-2 activation in the hepatic cells	[222]
		Naringenin and its β-cyclodextrin formulation	Swiss mice	- ↓ AST, ALT - ↑ CAT, SOD, GPx - ↓ MDA - improved centrilobular necrosis, steatosis, fibrosis, GSH, and the altered ultrastructure of hepatocytes	[223]
Flavonolignans	Silibinin	Collagenase type I and silibinin in chondroitin sulfate-coated multilayered nanoparticles	Kunming mice	- inhibited the development of hepatic fibrosis - ↓ the number of highly proliferative HSCs, the extent of collagen fiber deposition, and the number of inflammatory lesions - ↓ ALT, AST, TBIL - ↑ ALB - ↓ HYP - ↑ GSH-Px	[224]
	Silybin	Combined amphiphilic silybin meglumine nanosuspension	HepG2, LO2, LX-2, and RAW_264.7_ cells Kunming mice	- inhibited Col-1 secretion in LX-2 cells - exerted strong anti-fibrotic effects, by reducing deposition of collagen fibers and ECM along the central venous or portal area - prevented hepatic fibrosis by reversing HSC activation - ↓ α-SMA, Col-1, TGF-β	[225]
	Sylimarin	Silymarin-chitosan nanoparticles	Sprague-Dawley rats	- ↑ body weight - ↓ liver weight and liver index values - ↓ AST, ALT, ALP, TBIL - ↑ ALB - ↓ MDA, TGF-β - ↑ Nrf2 - ↑ the hepatic expression of protective miRNAs: miR-22, miR-29c, and miR-219a expression - ↓ TGFβ - R1, TGFβ - R2, Col3A1 - improved hepatic architecture - ↓ the degree of liver fibrosis - ↓ α-SMA	[226]
		Sylimarin–HPBCD and Sylimarin–RAMEB complexes	CD1 mice	- ↓ oxidative damage and ↑ antioxidant enzyme activities - alleviated CCl_4_-induced structural changes in liver - ↓ the score of liver fibrosis - ↓ NF-κB signaling and inflammatory cytokines: NF-κB p50, NF-κB p65, TNF-α, and IL-6 - inhibited activation and proliferation of HSCs - ↓ α-SMA - ↓ TGF-β1/Smad signaling pathway - ↓ TGF-β1, Smad 2 and Smad 3 - ↑ Smad 7 - ↓ Col-1 and the deposition of collagen in hepatic tissue - ↓ MMP-2, MMP-9, TIMP-1 - ↑ MMP-1	[227]
		Silymarin-loaded Eudragit^®^ RS100 nanoparticles	Wistar albino rats	- ↓ AST, ALT, TBIL - ↓ MDA - ↑ GSH - ↓ α-SMA - ↓ HYP, TGF-β1, TNF-α - ↓ the hepatic expression of TIMP-1 and CK-19 - ↑ HGF - ↑ the hepatic expression of MMP-2 - ↑ MMP-2/TIMP-1 ratio - ↓ collagen - restored hepatic architecture	[228]
Curcuminoids	Curcumin	Curcumin/chitosan-coated green-silver nanoparticles	Mice	- ↓ hepatic architectural lesions - restored the normal amount of collagenous connective tissue - modulated Col1A1, α-SMA, PDGFRB, TIMP-1, and ACTB gene expression - exerted its inhibitory role through the direct binding to fibrosis mediating proteins such as PDGFRB, TIMP-1, TLR-9, and TGF-β	[229]
		Curcumin–chitosan nanoparticles	Albino mice	- ↓ ALT, AST, ALP - ↓ AFP, caspase-3 - ↓ MDA - ↑ GSH, CAT	[230]
		Curcumin-loaded CTPP-PEG-PCL self-assembled micelles	Sprague-Dawley rats and Balb/c mice	- inhibited the liver fibrosis - ↓ ALT, AST - ↓ the relative positive area of liver fibrosis - enhanced the bioavailability of curcumin and extended the retention time of curcumin in vivo - enhanced the anti-fibrosis effect	[231]
		Curcumin with phosphatidylserine-decorated nanoparticles	Sprague-Dawley rats	- ↓ ALT, AST - ↓ the serum levels of pro-inflammatory cytokines TNF-α, IL-1b, and IL-6 - ↓ the degree of liver fibrogenesis - ↓ Col-1 - ↓ α-SMA - ↑ HGF expression and activated MMP-2 secretion in the liver	[232]
		Curcumin-zein nanospheres	SWR mice	- ↓ AST, ALT, ALP - ↑ ALB - ↓ pericentral and periportal fibrosis, inflammatory cells, ballooning degeneration, and steatosis - ↓ MDA - ↑ SOD, CAT, GSH-Px - ↓ Col-1, TIMP-2, TGF-β - ↑ MMP-2 - ↓ HYP	[233]
		Curcumin-loaded solid lipid nanoparticles	Wistar rats	- attenuated histopathological changes - improved the state of steatosis, ameliorated inflammation - ↓ AST, ALT - attenuated oxidative stress: - ↓ MDA - ↑ SOD - ↓ TNF-α	[234]

Legend: ↑ increased/up-regulated; ↓ decreased/down-regulated; ACTB, housekeeping beta-actin; AFP, alpha fetoprotein; ALB, albumin; ALP, alkaline phosphatase; ALT, alanine aminotransferase; AST, aspartate aminotransferase; CAT, catalase; CCl_4_, carbon tetrachloride; CHR, chrysin; CK-19, cytokeratin-19; Col IV, collagen IV; Col-1, collagen 1; Col1A1, collagen type I alpha 1; Col3A1, collagen type 3 alpha 1; CTPP-PEG-PCL, 3-carboxypropyl-triphenylphosphonium bromide-poly(ethylene glycol)-poly(3-caprolactone); ECM, extracellular matrix; GPx, glutathione peroxidase; GSH-Px, glutathione peroxidase; HA, hyaluronic acid; HGF, growth factor; HPBCD, hydroxypropyl β-cyclodextrin; HSCs, hepatic stellate cells; HYP, hydroxyproline; IL-1b, interleukin-1b; IL-6, interleukin-6; LN, laminin; LX-2, human hepatic stellate cell line; MDA, malondialdehyde; miRNAs, microRNAs; MMP-1, matrix metalloproteinase 1; MMP-2, matrix metalloproteinase 2; MMP-3, matrix metalloproteinase 3; MMP-9, matrix metalloproteinase 9; MSCs, mesenchymal stem cells; NF-κB, nuclear factor kappa B; Nrf2, nuclear factor erythroid 2-related factor 2; ORAC, oxygen radical absorbance capacity; PCIII, procollagen type III; PDGFRB, platelet-derived growth factor receptor beta; PLGA, poly lactic-co-glycolic acid; pro-MMP-2, pro-matrix metalloproteinase-2; RAMEB, randomly methylated β-cyclodextrin; SOD, superoxide dismutase; TBIL, total bilirubin; TGF-β1, transforming growth factor beta 1; TGFβR1, transforming growth factor beta-receptor 1; TGFβR2, transforming growth factor beta-receptor 2; TIMP-1, tissue inhibitor of metalloproteinases 1; TIMP-2, tissue inhibitor of metalloproteinases 2; TLR-9, toll-like receptor 9; TNF-α, tumor necrosis factor alpha; α-SMA, alpha smooth muscle actin.

## Data Availability

The data presented in this study are available on request from the corresponding authors.
